# Supplementary material for: Preferences for accessing sexual health services among middle-aged and older adults in the UK: a study protocol for a discrete choice experiment using mixed methods
Source: BMJ Open. 2023 May 8;13(5):e066783. doi: 10.1136/bmjopen-2022-066783 (PMC10174011; doi:10.1136/bmjopen-2022-066783)
Supplement: Supplementary data [file bmjopen-2022-066783supp001.pdf]

Appendix I

Sexual health in middle age and older adults in the UK
Interview Topic Guide

Date of interview:

Respondent ID:

Social Demographics

Sex:

Age:

Ethnic group:

Local County:

Do you consider yourself to have a disability?

Introduction (to be read by interviewer just before commencing the interview):

Thank you for accepting to take part in this interview. I would just like to reiterate that everything you say in the interview is confidential. All data collected will be anonymized. The interview itself will be open ended and the questions themselves are usually broad. There aren't any right or wrong answers. I'm simply interested in your experience and your views about sexual health services. Sexual health is fundamental to the overall wellbeing of many individuals. Is there anything you'd like to ask me?

Part I: Sexual health services

1. Have you used sexual health services in the last (6-12months)?
2. If no, any reasons?
3. If yes, were you satisfied with the service received?
4. Overall, how would you rate sexual health services you received?
   <Very satisfied, Satisfied, Neutral, Dissatisfied, Very dissatisfied>
5. Do you feel sexual health services available to older adults are adequate?
6. Do you talk to your GP about sexual health concerns?
7. Does your GP bring up sexual health related discissions with you?
8. Do you feel comfortable posting or commenting on sexual health topics on social media?

Kpokiri E, *et al. BMJ Open* 2023; 13:e066783. doi: 10.1136/bmjopen-2022-066783

Part II: Decision making process

1. What factors impact on your decision to seek sexual health services?
2. What would you access sexual health services for? General information and advice? Sexual function? STI testing?
3. How would you prefer to access sexual health services? Face to face or via telephone consultation? Or just online information?
4. For sexual health issues would you prefer to see a doctor that is:
    a. Same sex as you?
    b. Same ethnic background as you? Or speaks same language as you?
    c. A junior doctor or Very senior/ advanced doctor
    d. Have a friend, relation, partner go with you?
5. Where would you rather go for sexual health services? GP, Hospital, Pharmacy, Online, Older people groups/organisations medical outreach?

Part III: Barriers and facilitators

1. What factors prevent you from accessing sexual health services?
2. How would you prefer to access sexual health services?
3. What factors would make sexual health services easy and comfortable for you?
4. Would you be willing to participate in an open call and share messages about sexual health? With whom might you consider or not consider sharing the messages with? If so, what possible means of sharing would you prefer?

Appendix II: Preliminary qualitative results

So far, we have recruited and conducted 9 participants for the qualitative interviews as part of the first phase for the DCE project. Below are some preliminary results.

Socio-demographic characteristics of participants

There was a fair balance in gender with 4 males and 5 female respondents. Respondents were aged between 45 and 73 years with an average age of 58 years. All respondents were heterosexuals with more than half in a married /civil relationship (n=5). Few others were living with partner (n=2) and either single (n=1) or divorced (n=1). Respondents were either white-British (n=4) or black/African/British/Caribbean (n=5). Only one participant reported living with a disability and two-thirds of respondents confirmed using sexual health services within the last one year. See Table 1 below:

Table 1: Socio-demographics of participants

**Characteristics** (n = 9)	**n (%)**
**Gender**	
Male	4 (44)
Female	5 (56)
**Age** (years)	
45-50	3 (66)
51-60	2 (22)
61-70	3(33)
> 70	1 (11)
**Relationship status**	
Married/civil relationship	5 (56)
Single	1(11)
Living with partner	2 (22)
Divorced	1 (11)
**Sexual orientation**	
Straight heterosexual	9 (100)
Homosexual gay/lesbian	0 (0)
Bi-sexual	0 (0)
**Ethnicity**	
White-British	4 (44)
Black/British/Afro/Caribbean	5 (56)
**Disability**	
Yes	8 (89)
No	1 (11)
**Used SH Service in last year?**	
Yes	6 (67)
No	3 (33)

Preferences for accessing sexual health services and sharing messages

Participants that had used sexual health services were generally satisfied with services received. Most persons would rather not speak to their GP about their sexual health concerns unless it was absolutely necessary. Community sexual health clinics are preferred over GP's. Many older adults have sexual health needs related to decline sexual function and will not share sexual health related messages openly but are willing to send individual messages privately. Clear information about availability of sexual health services is needed.

Table 2: Preferences for accessing sexual health services and sharing messages

Questions	Responses	Themes
Do you talk to your GP about sexual health concerns	No/ if there is a need to	Most persons do not feel comfortable talking to their regular GP about their sexual health needs
Would you feel comfortable sharing messages relating to sexual health topics on social media?	Majority – No<br>Can send message privately to family friend if needed	Respondents will not share sexual health related messages openly but are willing to send individual messages privately if there is a need
How would you prefer to access sexual health services?	Mixed responses- Face to face or online	Depending on the severity of the sexual health problem either face to face or an initial telephone/online consultation is preferred
What would you access sexual health services for?	Sexual function<br>STI testing<br>Contraception<br>General info/advice<br>Pre-menopausal concerns<br>Assisted reproduction	Many older adults have sexual health needs related to decline sexual function<br><br>There is need for clearer information on what services are available and how to access them
Where would you rather go for sexual health services?	Community NHS clinics, Not GP<br>Anonymous NHS staff<br>Online?	Community sexual health clinics are preferred over GP's.
What factors would you consider when accessing sexual health services?	Convenience, quality of service (professional), anonymity, discretion,	With sexual health quality and professional services is always key. A platform that is easy and convenient is also important

Appendix III: Draft Attributes and levels for ranking

**Attributes**	**Levels**
**Service Type**	
Service provider for consultation	• GP clinic<br>• Sexual health clinic<br>• Pharmacy<br>• Online Platform
Mode of delivery	• Face to face<br>• Telephone call<br>• Video conference<br>• Online chat function
Provider initiates discussion of sexual health	• Yes<br>• No
Anonymity	• Name-based<br>• Partially anonymous<br>• Totally anonymous
**Provider Characteristics**	
Gender ethnicity and age of health personnel	• Same gender, age range and ethnicity<br>• Different gender age range and ethnicity
Seniority of health personnel	• Fairly recently qualified provider<br>• Older, more established provider
Relationship with health personnel	• Familiar provider/ existing relationship<br>• New provider
Personnel with intersectional training (incl language on gender and sexual minorities, disabilities etc)	• With intersectional training<br>• Without intersectional training
**Service Experience**	
Who attends consultation with you	• Family, friend, or personal care aide<br>• Medical assistant<br>• No one
Provider privacy policy	• Reassures patient of privacy<br>• No specific mention of privacy
Accessibility	• Accessible buildings and disability-friendly equipment<br>• Older, not updated accessibility accommodations

Appendix IV: Sample DCE survey

Participant ID:

Please do not leave any blank questions. There are no wrong answers.

ELIGIBILITY

01. Are you currently 45 years or older?

1) ☐ Yes、2) ☐ No

02. Have you had sexual health problems at all in the past five years?

1) ☐ Yes、2) ☐ No

03. How would you rate your sexual satisfaction in the past five years?

1) ☐ Very good、2) ☐ Good、3) ☐ Fair、4) Poor

04. How long have you been living in the UK?

1) ☐ Less than 6 months、2) ☐ Greater than 6 months、3) ☐ Not currently living in the UK

A. DEMOGRAPHICS

A1. What is your current age：______years old?

A2. What is your current sex?

1) Male

2) Female

A1. What city are you currently living in?

A2. What is your highest level of education?

1) ☐ Elementary
2) ☐ Middle school
3) ☐ High school
4) ☐ vocational college
5) ☐ 4-year college
6) ☐ graduate school

A3. What is your current marital/relationship status (past 6 months)?

Kpokiri E, *et al. BMJ Open* 2023; 13:e066783. doi: 10.1136/bmjopen-2022-066783

1) ☐ Single, no significant partner
2) ☐ Currently living with significant partner
3) ☐ Married
4) ☐ Divorced
5) ☐ Widowed
7）☐ Other：________

A4. Would you be willing to use sexual health services?
1) Yes
2) No

A5. How would you rate your health in general (past 6 months)
1) ☐ excellent、2) ☐ very good、3) ☐ good、4) ☐ fair、5) ☐ poor

B. Medical history & healthcare utilisation

B1. Have you ever been told by a healthcare professional that you have heart disease, cancer, diabetes, ADHD, a learning disability, asthma, chronic pain, or other chronic disease?
1) ☐ Yes、2) ☐ No [skip to D4]

B2. **If yes**, which one(s)? Check all that apply.
1) ☐ Heart disease、2) ☐ Cancer、3) ☐ Diabetes、4) ☐ ADHD/learning disability、
5) ☐ Asthma、6) ☐ chronic pain、7) ☐ Other [go to D3, otherwise skip to D4]

B3. **If other,** what was it? _____________

B4. In the past 2 years, how often have you visited a doctor?

1) ☐ I have not visited a doctor in the past 2 years、2) ☐ 1-2 times per year、3) ☐ 1-2 times per half-year、4) ☐ 1-2 times per month、5) ☐ 1-2 times per week

B5. In the past 2 years, have you ever avoided going to the doctor even though you were ill or injured?

1) ☐ Yes、2) ☐ No

B6. What type of health insurance do you have?

1) ☐ None、2) ☐ Urban employee、3) ☐ Urban resident、4) ☐ New rural cooperative、5) ☐ Private

B7. Would you be interested in discussing spiritual issues with your healthcare provider?

1) ☐ Yes、2) ☐ No、3) ☐ Unsure

B8. Do you have a (physical?) disability?

B9. If so, why type of (physical) disability?

B10. Have you ever consulted a healthcare provider for sexual health services (e.g., impotence, STIs, genital pain)?

C: Sexual behaviours

C1. What is your sexual orientation?
1) ☐ Straight、2) ☐ Gay/homosexual、3) ☐ Bisexual、4) ☐ Other/uncertain

C2. Are you currently in a romantic sexual relationship?
1) ☐ Yes、2) ☐ No

C3. Have you ever had anal/oral/vaginal sex with another person?
1) ☐ Yes、2) ☐ No

C4. How old were you when you first had anal, oral, or vaginal sex? __________ years old

C5. How many individuals have you ever had anal, oral, or vaginal sex with?
1) ☐ 1、2) ☐ 2-3、3) ☐ 4-5、4) ☐ 6-10、5) ☐ 11-20 、6) ☐ 21-50、7) ☐ >50

C6. Have you ever dated or had relations with anyone who has ever forced you to have sex when you did not want to？
1) ☐ Yes、2) ☐ No

C7. In the past 3 months, have you had sex? (check all that apply)
1) ☐ Yes, vaginal sex、2) ☐ Yes, anal sex、3) ☐ Yes, oral sex、4) ☐ No sex in past 3 months

C8. In the last 3 months, how often did you use a condom or other barrier method during sex?
1) ☐ Every time、2) ☐ Most of the time、3) ☐ Sometimes、4) ☐ Never

C9. Have any of these reasons kept you from getting tested for STI's (sexually transmitted infections)? If there's more than one reason, check all that apply
- ☐ You have never had sex
- ☐ It is too expensive
- ☐ You are afraid of what people might think
- ☐ You do not think you are at risk

Kpokiri E, *et al. BMJ Open* 2023; 13:e066783. doi: 10.1136/bmjopen-2022-066783

☐ You do not want to know if you have something
☐ You do not know where to go to get tested
☐ You do not know what is involved in getting tested
☐ You do not know what it means to have a positive test
☐ Other (Specify): ________________________________

D: Sexual health services Discrete choice Experiment

The following questions will present three sexual health service scenarios (Choice 'A' and 'B'), and a "Do not seek sexual health services" scenario (Choice 'C'). *Assuming that you have a need for sexual health services [examples or definitions inserted here] , which sexual health doctor consultation scenario would you most prefer? A,B, or C?* ***. There are no incorrect answers.***"

D1. Please choose the scenario that you prefer the most

Scenario A	Scenario B	Scenario C
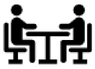<br>Face to face<br>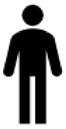<br>Alone with GP<br>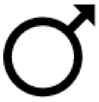<br>Male GP	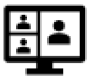<br>Teleconference consultation<br>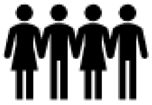<br>Visit GP with family or friends<br>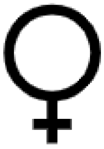<br>Female GP	Do not seek sexual health services

D2. Please choose the scenario that you prefer the most
…
D3. Please choose the scenario that you prefer the most
…
D4. Please choose the scenario that you prefer the most
…
D5. Please choose the scenario that you prefer the most
…
D6. Please choose the scenario that you prefer the most

Kpokiri E, *et al. BMJ Open* 2023; 13:e066783. doi: 10.1136/bmjopen-2022-066783

…

D7. Please choose the scenario that you prefer the most

…

D8. Please choose the scenario that you prefer the most

…
