# Supplementary material for: Preferences for accessing sexual health services among middle-aged and older adults in the UK: a study protocol for a discrete choice experiment using mixed methods
Source: BMJ Open. 2023 May 8;13(5):e066783. doi: 10.1136/bmjopen-2022-066783 (PMC10174011; doi:10.1136/bmjopen-2022-066783)
Supplement: Author's manuscript [file bmjopen-2022-066783.draft_revisions.pdf]

BMJ Open is committed to open peer review. As part of this commitment we make the peer review history of every article we publish publicly available.

When an article is published we post the peer reviewers' comments and the authors' responses online. We also post the versions of the paper that were used during peer review. These are the versions that the peer review comments apply to.

The versions of the paper that follow are the versions that were submitted during the peer review process. They are not the versions of record or the final published versions. They should not be cited or distributed as the published version of this manuscript.

BMJ Open is an open access journal and the full, final, typeset and author-corrected version of record of the manuscript is available on our site with no access controls, subscription charges or pay-per-view fees (http://bmjopen.bmj.com).

If you have any questions on BMJ Open's open peer review process please email info.bmjopen@bmj.com

BMJ Open

# Preferences for accessing sexual health services among older adults including those with disabilities in the UK: A Discrete Choice Experiment Protocol


| | |
|---|---|
| Journal: | *BMJ Open* |
| Manuscript ID | bmjopen-2022-066783 |
| Article Type: | Protocol |
| Date Submitted by the Author: | 25-Jul-2022 |


| | |
|---|---|
| Complete List of Authors: | Kpokiri, Eneyi; London School of Hygiene and Tropical Medicine Faculty of Infectious and Tropical Diseases, Department of Clinical Research<br>Pan, Stephen W.; Xi'an Jiaotong-Liverpool University, Department of Health and Environmental Sciences<br>Ong , Jason ; Monash University; London School of Hygiene & Tropical Medicine<br>Greaves, Emily; London School of Hygiene & Tropical Medicine<br>Khan, Junead; London School of Hygiene & Tropical Medicine<br>Bowen, Sophie; London School of Hygiene and Tropical Medicine Faculty of Infectious and Tropical Diseases, Department of Clinical Research<br>Jannaway, Tracey; Independent Living Alternatives<br>Terris-Prestholt, Fern; London School of Hygiene and Tropical Medicine<br>Tanton, Clare; London School of Hygiene and Tropical Medicine, Global Health & Development<br>Kuper, Hannah; The London School of Hygiene & Tropical Medicine, Clinical Research<br>Shakespeare, Tom; London School of Hygiene and Tropical Medicine Faculty of Infectious and Tropical Diseases<br>Tucker, Joseph; University of North Carolina Project China; London School of Hygiene & Tropical Medicine, Faculty of Infectious and Tropical Diseases<br>Wu, Dan; Sichuan University West China School of Public Health Department of Epidemiology and Health Statistics; London School of Hygiene & Tropical Medicine, Faculty of Infectious and Tropical Diseases |


| | |
|---|---|
| Keywords: | Sexual dysfunction < UROLOGY, SEXUAL MEDICINE, GERIATRIC MEDICINE |
| | |


SCHOLARONE™ Manuscripts

# Preferences for accessing sexual health services among older adults including those with disabilities in the UK: A Discrete Choice Experiment Protocol

*Eneyi E. Kpokiri[1], *Stephen W. Pan[2], Jason J. Ong[1,3], Emily Greaves[1], Junead Khan[1], Sophie Bowen[1], Tracey Jannaway[4], Fern Terris-Prestholt[5], Clare Tanton[5], Hannah Kuper[1], Tom Shakespeare[1], Joseph D. Tucker[1,6], Dan Wu[1,7]

[1] Department of Clinical Research, London School of Hygiene and Tropical Medicine, London, United Kingdom

[2] Department of Health and Environmental Sciences, Xi'an Jiaotong-Liverpool University, Suzhou, China.

[3] Central Clinical School, Monash University, Melbourne, Australia

[4] Independent Living Alternatives, London, United Kingdom

[5] Faculty of Public Health & Policy, London School of Hygiene & Tropical Medicine, London, United Kingdom

[6] Division of Infectious Diseases, Department of Medicine, University of North Carolina at Chapel Hill, Chapel Hill, NC, United States

[7] Department of Epidemiology, West China School of Public Health, Sichuan University, Chengdu, China

*These authors contributed equally

Corresponding author:

Dan Wu, Department of Epidemiology, West China School of Public Health, Sichuan University, Chengdu, China

Department of Clinical Research, London School of Hygiene and Tropical Medicine, London, United Kingdom

Email: dan.wu@lshtm.ac.uk



For peer review only - http://bmjopen.bmj.com/site/about/guidelines.xhtml

## ABSTRACT

**Introduction:** Sexual health is essential for general health and wellbeing, including for older adults. Sexual health services for older adults, especially those with disabilities, are not prioritized and optimizing available services for this population is often overlooked. Not much is known about preferences for accessing sexual health services among older people or level of satisfaction with current services. The aim of this study is to access preferences for seeking sexual health services among older adults, including those with disabilities, in the UK. The study will utilise Discrete Choice Experiments (DCEs), which have been used as a tool to explore preferences in various health service delivery.

**Methods and Analysis:** First, we will conduct in-depth semi structured interviews with 20-30 older adults (aged 45+), including older adults with disabilities and those from sexual minority groups. Interviews will explore indications, preferences and factors related to accessing sexual health services. Themes and sub-themes emerging from the analysis of the interviews will then be used to design the choice sets and attribute level for the DCEs. Secondly, for the DCEs, we will design choice sets composed of sexual health service delivery scenarios. The software NGENE will be used to develop the experimental design matrix for the DCE. We will use descriptive statistics to summarize the key sociodemographic characteristics of the study population. Multinomial logit (MNL), latent class (LC) and mixed logit (MXL) models will be explored to assess sexual health service preferences and preference heterogeneity.

**Ethics and Dissemination:** Ethical approval for both parts of this study was granted by the Research and Ethics Committee at the London School of Hygiene and Tropical Medicine, United Kingdom (26134). Findings from this study will be disseminated widely to relevant stakeholders via scheduled meetings, webinars, presentations at conferences and in peer reviewed journal manuscripts.


### Strengths and Limitations of this study

- This is the first DCEs exploring preferences for sexual health services among older adults including those with disabilities.
- Our DCE is designed based on detailed formative research whereby choice sets are developed from emerging themes from 21 semi-structured interviews



For peer review only - http://bmjopen.bmj.com/site/about/guidelines.xhtml

- While the survey targets mature adults (age 45 and older), we sought to ensure inclusion of those from sexual minority groups and those with disabilities by recruiting from local community-based organisations. This is to ensure diversity within this population to allow for sub-analysis
- Recruitment will be restricted to only persons within the UK as a result, generalising the findings may be restricted to only the UK or other places with similar settings.

**Background**

Sexual health is an integral part of health, wellbeing, and quality of life. However, many existing sexual health programmes, interventions and research are tailored for youth and younger adults.[1] The sexual health needs of older adults are often neglected, including by health professionals.[2] There are also a host of diverse challenges and barriers that interplay in how and why this age group access sexual health services. Consequently, there is suboptimal uptake of sexual health services among middle aged and older adults.[3,4]Within the broader group of "older people", certain groups may be particularly neglected, such as older people with disabilities, as they face additional barriers to accessing health services.[5] This is an important issue, as almost one in five people in the UK are aged 65+, and 42% of people in this age group report a disability.[6] Disability is defined under the Equality act of 2010 as having a physical or mental impairment that has substantial and long-term negative effect on the ability to do normal daily activities.[7] Despite the growing population of older adults and an increasing body of evidence suggesting sexual health is important to older adult quality of life, there is limited evidence to support preferences and tailor services for specific groups within this population.[8,9]

This study will explore preferences for seeking sexual health services among older adults. This is part of a larger study to inform areas for improved sexual health services for older adults, including those with disabilities, in the United Kingdom. Our study findings will identify issues and generate new knowledge on the preferences for using sexual health services among older adults in the UK.

Discrete choice experiments (DCEs) are a quantitative approach to measure strength of preferences. It is an attribute-based measure of utility, based on the assumptions that healthcare interventions, services or policies, can be described by their attributes and that user preferences depend upon the levels of these attributes.[10] DCEs ask participants to make choices from hypothetical alternatives – GIVE AN EXAMPLE. They are increasingly used in


For peer review only - http://bmjopen.bmj.com/site/about/guidelines.xhtml

health research to study patient and/or physician preferences.[11] For instance, DCEs have been used to determine preferences for HIV prevention and testing among a range of specific populations and settings to inform strategies for optimising health services.[12-17] DCEs have also been employed to study preferences for social care among older adults.[18] However, to date, no DCE studies have been conducted to determine preferences for sexual health services among older adults. Our study will use DCEs to explore preferences for accessing sexual health services and any heterogeneity that exists among older adults in the UK.

**Study aims and objectives**

This study aims to explore preferences for seeking sexual health services among older adults, including sexual minorities and those with disabilities, in the UK. Specific objectives include 1) To use DCE to identify preferences related to accessing services for sexual health 2) To explore the heterogeneity among older adults' preferences for utilization of sexual health services, particularly by disability status.

**Methods and Analysis**

**Overview of approach and methods**

The research will be conducted in two phases. First, we will organise semi-structured interviews with older adults to better understand their preferences and the importance of sexual health service and message sharing attributes in influencing their use of these services. Interview data will be analysed thematically, and emerging themes will inform which attributes and levels will be included in the choice sets for the DCEs.

**Patient and Public Involvement**

Community representatives have been engaged in the study design, and will be continuously engaged in our survey distribution and participant recruitment, data interpretation, reporting, or dissemination plans of our research.

**Phase I- Semi-structured interviews**

First, we will conduct between 20 to 30 semi-structured individual interviews with older adults and stop when data saturation is reached. This formative qualitative research will identify factors that are considered in the decision-making processes among this population related to sexual health services (e.g., awareness, location, access process, quality of care or



For peer review only - http://bmjopen.bmj.com/site/about/guidelines.xhtml

qualifications of healthcare providers, cost), identifying barriers and facilitators to service utilisation and sexual health message sharing based on older adults' perceptions of and/or experiences with sexual health services and interaction with sexual health messages. We will also field test the questionnaire using cognitive interviews prior to official start to help us understand how older individuals mentally process and respond to survey questions and DCE choice sets.

Participants will be purposively selected across different locations, age range, ethnic backgrounds, and disability status. Recruitment will be via community-based organisations designed for older adults. Interviews will be conducted via telephone calls or teleconference using Zoom, Skype, or Microsoft Teams and facilitated by an experienced qualitative researcher. Teleconference calls may be either audio or video according to participant preferences. Interviews will be conducted using a topic guide (See Appendix I) and we will aim for approximately 30 minutes per interview session. Adjustments will be made as needed to promote the inclusion of persons with disabilities (e.g., use of sign language interpretation, simple language, inclusion of breaks). Each participant will receive an honorarium worth £30 in appreciation for their participation. Interviews will be audio-recorded following participant consent and transcribed verbatim prior to analysis.

We will undertake thematic analysis of the qualitative interview data. A codebook will be developed both inductively and deductively to guide analysis. Outcomes of qualitative data analysis will be the indications and use of sexual health services, preferences and factors affecting older adults' decision making to seek intimate and/or sexual relationships, barriers and facilitators to sexual health service utilisation which will inform the DCE survey (See Appendix II for some preliminary results).

**Phase II- Discrete Choice Experiment survey**

Second, we will use the qualitative information from Phase I to inform what attributes (characteristics) and levels (choices) will be included in the unlabeled choice set, which will be administered in an online survey to a sample of older adults to identify their preferences for accessing sexual health services. We will design choice sets composed of hypothetical sexual health service delivery scenarios. In addition, we will aim to recruit people with disabilities and people from sexual minority groups for the survey to allow sub-analyses of the results.



For peer review only - http://bmjopen.bmj.com/site/about/guidelines.xhtml

## Developing the DCE design

*Pictorial representation of attribute levels and DCE choice set size*

A ranking exercise will be organized with some of the interview participants to align and finalise the attributes and levels that will be included in the final choice sets (Appendix III). After attributes and levels have been finalised, we will generate visual graphics to represent each attribute level in the DCE survey (See Appendix IV for a sample). To ensure that the visual graphics effectively represent the intended meaning of each attribute level (i.e., construct validity), we will conduct qualitative think-aloud exercises with 3-5 individuals from the target population, and participants will provide feedback about how they interpret and feel about the graphics.[19] This will be an iterative process with the draft pictorial concepts being tested until they are appropriately representing the intended concept. DCE surveys tend to present between 4 and 18 choice sets. During these interviews, we will also explore the optimal number of choice sets to present to each respondent to manage potential cognitive fatigue. Similar to the phase 1 interviews, participants will be purposively selected across different locations, age range, ethnic backgrounds and disability status. Again, reasonable accommodation will be made to promote inclusion of persons with disabilities, although people with severe cognitive/intellectual impairment will not be included in this step. Recruitment will be through collaborations with community-based organisations designed for older adults with which our research team already have existing research collaborations with.

*Experimental design*

After visual graphics of the attribute levels have been finalised, we will use the software NGENE to develop the experimental design matrix for the DCE pilot.[20] For the pilot test, no assumptions about the parameter estimates will be made. We will then specify the appropriate number of attributes, levels, alternatives, blocks, and choice sets, and NGENE will iteratively generate design matrices of increasing statistical efficiency. We will use the design with the lowest D-error generated by NGENE. An example of a choice set and DCE design is presented in Figure 1. Graphics will have alt-text enabled so that they can be accessed by people with visual impairments.

*Blocking and randomisation of choice set ordering*



For peer review only - http://bmjopen.bmj.com/site/about/guidelines.xhtml

Depending on the total number of choice sets in the DCE design, choice sets may be grouped into blocks. Respondents will then be randomly assigned to one of the blocks and asked to complete all choice sets in their block. We anticipate using between 4-10 blocks, with each block containing 4-10 choice sets. To guard against potential ordering effects, the order of the choice sets within each block will be randomised for each participant.

*Pilot testing*

We intend to use convenience sampling to recruit up to 30 participants for the pilot test. Participants in the pilot test will receive a £20 gift card for completing the survey. Conducting the pilot test will serve two purposes: (1) identify potential problems with the DCE and survey in general (e.g., cognitive fatigue or incoherent questions or lack of accessibility) and (2) generate priors (i.e., parameter estimates) that will be used to develop the design of the final full-scale DCE. We will analyse the pilot data with multinomial logit models (MNL), given the limited sample size. Priors from the pilot test will be input into NGENE to finalise and enhance the efficiency of the full-scale DCE design.

*Full-scale DCE survey*

Community-based organizations, local clinics, GP practices and older people care homes will be contacted to help distribute the online survey for older adults. Recruitment will be carried out purposively from urban and suburban areas, evenly spread across the middle age and older adults age groups. We will create formats for completing the DCE surveys both online and offline.

**Eligibility criteria:**

We will recruit persons who meet all of the following criteria:

1) Aged 45 years or older

2) Have had sexual health problems, low sexual satisfaction or function in the last five years

3) Resident in the UK for at least six months

4) Able to consent to participation.

With consent, participants of the DCE will be entered into a draw where up to 20 participants will be randomly selected to receive a £100 gift card.



For peer review only - http://bmjopen.bmj.com/site/about/guidelines.xhtml

*Quality assessment*

It is possible that some **participants** known as “speeders” may try to complete the DCE survey as soon as possible without seriously considering the survey questions. “Speeders” will be identified as those who complete the survey at least 50% faster than the median time for survey completion among all valid survey respondents. We will then conduct a sensitivity analysis and determine whether or not the removal of identified speeders significantly alters the results.

We will also assess the possibility of order effect bias by introducing a dummy variable for the order of the choice set. We will test for left and right bias by including a constant for the left choice and examining the extent to which the left position of the alternatives in the choice sets influences the likelihood of being chosen. Comparable probabilities of the left and centre alternatives being selected would suggest that the absence of bias.

**Sample size calculation**

Given the absence of any DCE priors for sexual health preferences of older adults in the UK, we used the following parametric-based DCE sample size calculation formula

$$n \geq \frac{(1-p)}{rpa^2} \times \left[ (\Phi^{-1}\left(1 - \frac{\alpha}{2}\right) \right]^2$$

**Equation 1: Parametric DCE sample size formula[21]**

Whereby $n$ is the minimum sample size needed to detect $p$, the assumed true population probability of selecting uptake of sexual health services over “opt-out”, and whereby $r$ is the number of choice sets that each individual completes, $a$ is the level of precision around the assumed population probability, $\Phi^{-1}$ is the inverse cumulative normal distribution function, and $\alpha$ is the significance level. Conservatively assuming that the true population probability of sexual health service uptake is 0.5 and assuming that each participant will complete eight choice sets, we estimate that a sample of 191 participants will be needed to generate a point estimate ± 0.05 of the true population probabilities and with 95% confidence. However, to maintain power for two-group subgroup analyses, we will aim to recruit a total **240** participants including those from sexual minorities groups and those with disabilities.

**Statistical analysis**



For peer review only - http://bmjopen.bmj.com/site/about/guidelines.xhtml

We will use descriptive statistics to summarize the key sociodemographic characteristics of the study population. We will assess sexual health service preferences and preference heterogeneity using multinomial logit (MNL), latent class (LC) and mixed logit (MXL) models. While the MNL is the starting point for most discrete choice models, it suffers from some unrealistic assumptions, particularly around unobserved heterogeneity. The MXL model can accommodate unobserved heterogeneity. The MXL analysis will be conducted with1000 Halton draws and will assume normally distributed preference heterogeneity for each attribute level. We will assess for unobserved heterogeneity by examining the statistical significance of the standard deviations of the random parameters in the MXL model. We will calculate the relative importance and predicted probabilities of opt-out for select sexual health service scenarios. We will assess how sexual health service and message sharing preferences could vary among those willing and unwilling to use sexual health services by using interaction terms for sociodemographic characteristics.

We will perform latent class analyses to elucidate if there are unobserved groups of people with similar preferences for use of sexual health service.[22] First, we will conduct a latent class conditional logit model that includes individual characteristics and attribute level preferences.[22] Selection of individual characteristics will be determined by expert opinion and findings from the formative qualitative interviews. Second, we will determine the optimal number of subgroups by comparing the BIC values of 2, 3, 4, 5, 6, and 7-class models and each models' interpretability and class size distribution.[22] Third, we will use descriptive statistics to characterise each class based on their members' preferences and characteristics. All analyses will use effects coding and be weighted to reflect the population of older adults in the UK.

*Attribute relative importance and probability of opt-out*

For each of the sex-class combinations, the relative importance of each attribute will be calculated by dividing the range of the parameter estimates of a given attribute by the summation of the ranges of all attributes.[23]

We will further illustrate potential preference heterogeneities by using equation 2 to calculate the predicted probabilities of selecting "opt-out" over select sexual health service scenarios for each sex-class combination. Specifically, the predicted probability of a participant choosing alternative *i* over alternative *j* can be estimated by dividing the exponentiated



For peer review only - http://bmjopen.bmj.com/site/about/guidelines.xhtml

summation of coefficients for scenario *i* by the exponentiated summation of coefficients for scenario *j*.

$$\text{Pi} = \frac{e^{\beta x_i}}{\sum e^{\beta x_j}} \quad \text{Pi} = \frac{e^{\beta x_i}}{\sum e^{\beta x_j}}$$

**Equation 2: Predicted probability of selecting alternative *i* within a two-alternative choice set**

**Impact**

This study will improve our understanding of older people's preferences and factors important for sexual health services. Older adults' preferences will be useful to develop sexual health services and programs tailored for older people.

**Feasibility**

Our project is feasible as the team has expertise from multiple disciplines to examine sexual health among older adults with unique strengths in DCEs, disability studies, and sexual health research.

**Funding**

The study is supported by Economic and Social Research Council, UK Research and Innovation (UKRI) [grant number: ES/T014547/1].

**Ethical approval**

Ethical approval was obtained from the Research and Ethics Committee at the London School of Hygiene and Tropical Medicine, United Kingdom (26134)

**Competing interests**

None.

**Contributors Statement**

DW and JDT conceived the idea. DW oversees the study. EEK and SWP wrote the first draft of the protocol paper. EEK, EG and SB conducted the interviews, developed attributes and levels. JJO, SWP, FTP and JDT provided additional advice on the discrete choice experiment design and will carry out the experiment design. TJ, CT, HK, TS and JDT provided expertise

For peer review only

advice on improving inclusiveness and appropriateness of the protocol. All authors reviewed, provided constructive comments on improving the design and the manuscript. All authors have seen and approve the final version of this manuscript.

**References:**


1. Ezhova I, Savidge L, Bonnett C, Cassidy J, Okwuokei A, Dickinson T. Barriers to older adults seeking sexual health advice and treatment: A scoping review. *International journal of nursing studies*. 2020;107:103566.
2. McGrath M, Lynch E. Occupational therapists' perspectives on addressing sexual concerns of older adults in the context of rehabilitation. *Disability and rehabilitation*. 2014;36(8):651-657.
3. Gott M, Hinchliff S, Galena E. General practitioner attitudes to discussing sexual health issues with older people. *Social science & medicine*. 2004;58(11):2093-2103.
4. Beaudreau SA, Van Moorleghem K, Dodd SM, Liou-Johnson V, Suresh M, Gould CE. Satisfaction with a vacuum constriction device for erectile dysfunction among middle-aged and older veterans. *Clinical Gerontologist*. 2021;44(3):307-315.
5. Organization WH. *Summary: World report on disability 2011*. 2011. 6099570705.
6. GOV.UK. Family Resources Survey: financial year 2020 to 2021. Accessed 14 June, 2022. https://www.gov.uk/government/statistics/family-resources-survey-financial-year-2020-to-2021/family-resources-survey-financial-year-2020-to-2021#disability-1
7. GOV.UK. Equality Act 2010. GOV.UK. Accessed 02 March 2022, 2022. https://www.gov.uk/definition-of-disability-under-equality-act-2010#:~:text=You're%20disabled%20under%20the,to%20do%20normal%20daily%20activities.
8. Lindau ST, Schumm LP, Laumann EO, Levinson W, O'Muircheartaigh CA, Waite LJ. A study of sexuality and health among older adults in the United States. *New England Journal of Medicine*. 2007;357(8):762-774.
9. Hinchliff S, Tetley J, Lee D, Nazroo J. Older adults' experiences of sexual difficulties: qualitative findings from the English Longitudinal Study on Ageing (ELSA). *The Journal of Sex Research*. 2018;55(2):152-163.
10. Ryan M, Gerard K. Using discrete choice experiments to value health care programmes: current practice and future research reflections. *Applied health economics and health policy*. 2003;2(1):55-64.
11. Quaife M, Terris-Prestholt F, Di Tanna GL, Vickerman P. How well do discrete choice experiments predict health choices? A systematic review and meta-analysis of external validity. *The European journal of health economics*. 2018;19(8):1053-1066.
12. Strauss M, George G, Lansdell E, et al. HIV testing preferences among long distance truck drivers in Kenya: a discrete choice experiment. *AIDS care*. 2018;30(1):72-80.
13. Ong JJ, Nwaozuru U, Obiezu-Umeh C, et al. Designing HIV Testing and Self-Testing Services for Young People in Nigeria: A Discrete Choice Experiment. *The Patient-Patient-Centered Outcomes Research*. 2021:1-12.
14. Schaffer EM, Gonzalez JM, Wheeler SB, Kwarisiima D, Chamie G, Thirumurthy H. Promoting HIV testing by men: a discrete choice experiment to elicit preferences and




For peer review only - http://bmjopen.bmj.com/site/about/guidelines.xhtml

predict uptake of community-based testing in Uganda. *Applied health economics and health policy*. 2020:1-20.
15. Ong JJ, Nwaozuru U, Obiezu-Umeh C, et al. Designing HIV Testing and Self-Testing Services for Young People in Nigeria: A Discrete Choice Experiment. *The Patient-Patient-Centered Outcomes Research*. 2021;14(6):815-826.
16. Pan SW, Durvasula M, Ong JJ, et al. No place like home? Disentangling preferences for HIV testing locations and services among men who have sex with men in China. *AIDS and Behavior*. 2019;23(4):847-859.
17. Terris-Prestholt F, Mulatu A, Quaife M, et al. Using choice experiments to improve equity in access to socially marketed HIV prevention products. *Journal of choice modelling*. 2021;41:100319.
18. Ryan M, Netten A, Skåtun D, Smith P. Using discrete choice experiments to estimate a preference-based measure of outcome—an application to social care for older people. *Journal of health economics*. 2006;25(5):927-944.
19. Aday LA, Cornelius LJ. *Designing and conducting health surveys: a comprehensive guide*. John Wiley & Sons; 2006.
20. Metrics C. NGENE 1.1. 2 User Manual and Reference Guide: the cutting edge in experimental design. *USA: Choice Metrics*. 2014;
21. de Bekker-Grob EW, Donkers B, Jonker MF, Stolk EA. Sample size requirements for discrete-choice experiments in healthcare: a practical guide. *The Patient-Patient-Centered Outcomes Research*. 2015;8(5):373-384.
22. Zhou M, Thayer WM, Bridges JF. Using latent class analysis to model preference heterogeneity in health: a systematic review. *Pharmacoeconomics*. 2018;36(2):175-187.
23. Hauber AB, González JM, Groothuis-Oudshoorn CG, et al. Statistical methods for the analysis of discrete choice experiments: a report of the ISPOR conjoint analysis good research practices task force. *Value in health*. 2016;19(4):300-315.

For peer review only

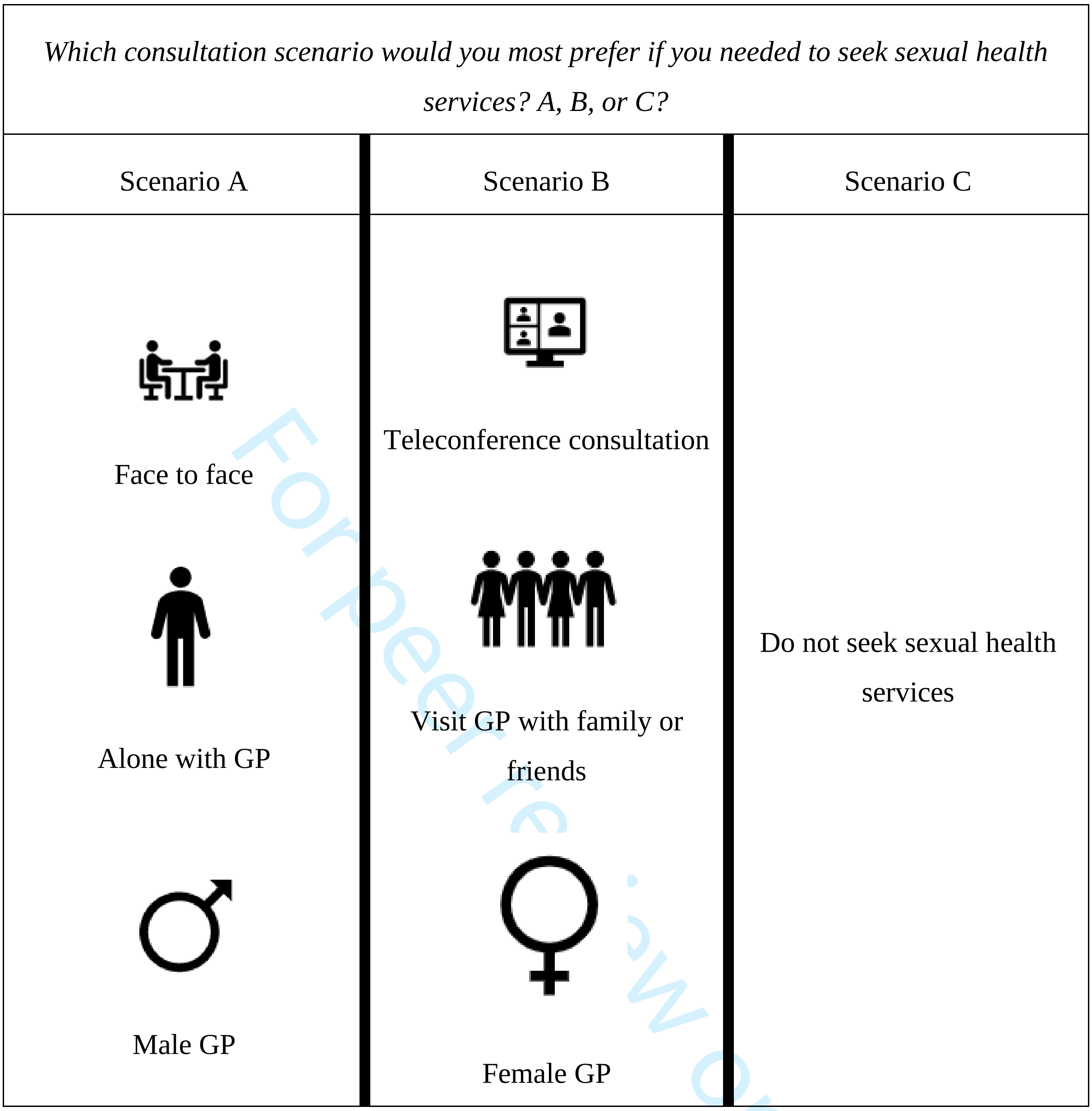


**Figure 1: Fictitious example of a choice set**

# Appendix I

## Sexual health in middle age and older adults in the UK
## Interview Topic Guide

Date of interview:

Respondent ID:

**Social Demographics**

Sex:

Age:

Ethnic group:

Local County:

Do you consider yourself to have a disability?

**Introduction (to be read by interviewer just before commencing the interview):**

Thank you for accepting to take part in this interview. I would just like to reiterate that everything you say in the interview is confidential. All data collected will be anonymized. The interview itself will be open ended and the questions themselves are usually broad. There aren't any right or wrong answers. I'm simply interested in your experience and your views about sexual health services. Sexual health is fundamental to the overall wellbeing of many individuals. Is there anything you'd like to ask me?

**Part I: Sexual health services**

1. Have you used sexual health services in the last (6-12months)?
2. If no, any reasons?
3. If yes, were you satisfied with the service received?
4. Overall, how would you rate sexual health services you received?
   <Very satisfied, Satisfied, Neutral, Dissatisfied, Very dissatisfied>
5. Do you feel sexual health services available to older adults are adequate?
6. Do you talk to your GP about sexual health concerns?
7. Does your GP bring up sexual health related discissions with you?
8. Do you feel comfortable posting or commenting on sexual health topics on social media?



For peer review only - http://bmjopen.bmj.com/site/about/guidelines.xhtml

**Part II: Decision making process**

1. What factors impact on your decision to seek sexual health services?
2. What would you access sexual health services for? General information and advice? Sexual function? STI testing?
3. How would you prefer to access sexual health services? Face to face or via telephone consultation? Or just online information?
4. For sexual health issues would you prefer to see a doctor that is:
    a. Same sex as you?
    b. Same ethnic background as you? Or speaks same language as you?
    c. A junior doctor or Very senior/ advanced doctor
    d. Have a friend, relation, partner go with you?
5. Where would you rather go for sexual health services? GP, Hospital, Pharmacy, Online, Older people groups/organisations medical outreach?

**Part III: Barriers and facilitators**

1. What factors prevent you from accessing sexual health services?
2. How would you prefer to access sexual health services?
3. What factors would make sexual health services easy and comfortable for you?
4. Would you be willing to participate in an open call and share messages about sexual health? With whom might you consider or not consider sharing the messages with? If so, what possible means of sharing would you prefer?

For peer review only

## Appendix II: Preliminary qualitative results

So far, we have recruited and conducted 9 participants for the qualitative interviews as part of the first phase for the DCE project. Below are some preliminary results.

### *Socio-demographic characteristics of participants*

There was a fair balance in gender with 4 males and 5 female respondents. Respondents were aged between 45 and 73 years with an average age of 58 years. All respondents were heterosexuals with more than half in a married /civil relationship (n=5). Few others were living with partner (n=2) and either single (n=1) or divorced (n=1). Respondents were either white-British (n=4) or black/African/British/Caribbean (n=5). Only one participant reported living with a disability and two-thirds of respondents confirmed using sexual health services within the last one year. See Table 1 below:

**Table 1: Socio-demographics of participants**

| **Characteristics** (n = 9) | **n (%)** |
|---|---|
| **Gender** | |
| Male | 4 (44) |
| Female | 5 (56) |
| **Age** (years) | |
| 45-50 | 3 (66) |
| 51-60 | 2 (22) |
| 61-70 | 3(33) |
| > 70 | 1 (11) |
| **Relationship status** | |
| Married/civil relationship | 5 (56) |
| Single | 1(11) |
| Living with partner | 2 (22) |
| Divorced | 1 (11) |
| **Sexual orientation** | |
| Straight heterosexual | 9 (100) |
| Homosexual gay/lesbian | 0 (0) |
| Bi-sexual | 0 (0) |
| **Ethnicity** | |
| White-British | 4 (44) |
| Black/British/Afro/Caribbean | 5 (56) |
| **Disability** | |
| Yes | 8 (89) |
| No | 1 (11) |
| **Used SH Service in last year?** | |
| Yes | 6 (67) |
| No | 3 (33) |


For peer review only - http://bmjopen.bmj.com/site/about/guidelines.xhtml

### *Preferences for accessing sexual health services and sharing messages*

Participants that had used sexual health services were generally satisfied with services received. Most persons would rather not speak to their GP about their sexual health concerns unless it was absolutely necessary. Community sexual health clinics are preferred over GP's. Many older adults have sexual health needs related to decline sexual function and will not share sexual health related messages openly but are willing to send individual messages privately. Clear information about availability of sexual health services is needed.

**Table 2: Preferences for accessing sexual health services and sharing messages**

| Questions | Responses | Themes |
|---|---|---|
| Do you talk to your GP about sexual health concerns | No/ if there is a need to | Most persons do not feel comfortable talking to their regular GP about their sexual health needs |
| Would you feel comfortable sharing messages relating to sexual health topics on social media? | Majority – No<br>Can send message privately to family friend if needed | Respondents will not share sexual health related messages openly but are willing to send individual messages privately if there is a need |
| How would you prefer to access sexual health services? | Mixed responses- Face to face or online | Depending on the severity of the sexual health problem either face to face or an initial telephone/online consultation is preferred |
| What would you access sexual health services for? | Sexual function<br>STI testing<br>Contraception<br>General info/advice<br>Pre-menopausal concerns<br>Assisted reproduction | Many older adults have sexual health needs related to decline sexual function<br><br>There is need for clearer information on what services are available and how to access them |
| Where would you rather go for sexual health services? | Community NHS clinics, Not GP<br>Anonymous NHS staff<br>Online? | Community sexual health clinics are preferred over GP's. |
| What factors would you consider when accessing sexual health services? | Convenience, quality of service (professional), anonymity, discretion, | With sexual health quality and professional services is always key. A platform that is easy and convenient is also important |



For peer review only - http://bmjopen.bmj.com/site/about/guidelines.xhtml

## Appendix III: Draft Attributes and levels for ranking

| Attributes | Levels |
|---|---|
| **Service Type** | |
| *Service provider for consultation* | • GP clinic<br>• Sexual health clinic<br>• Pharmacy<br>• Online Platform |
| *Mode of delivery* | • Face to face<br>• Telephone call<br>• Video conference<br>• Online chat function |
| *Provider initiates discussion of sexual health* | • Yes<br>• No |
| *Anonymity* | • Name-based<br>• Partially anonymous<br>• Totally anonymous |
| **Provider Characteristics** | |
| *Gender ethnicity and age of health personnel* | • Same gender, age range and ethnicity<br>• Different gender age range and ethnicity |
| *Seniority of health personnel* | • Fairly recently qualified provider<br>• Older, more established provider |
| *Relationship with health personnel* | • Familiar provider/ existing relationship<br>• New provider |
| *Personnel with intersectional training (incl language on gender and sexual minorities, disabilities etc)* | • With intersectional training<br>• Without intersectional training |
| **Service Experience** | |
| *Who attends consultation with you* | • Family, friend, or personal care aide<br>• Medical assistant<br>• No one |
| *Provider privacy policy* | • Reassures patient of privacy<br>• No specific mention of privacy |
| *Accessibility* | • Accessible buildings and disability-friendly equipment<br>• Older, not updated accessibility accommodations |



For peer review only - http://bmjopen.bmj.com/site/about/guidelines.xhtml

**Appendix IV: Sample DCE survey**

**Participant ID:**

Please do not leave any blank questions. There are no wrong answers.

**ELIGIBILITY**

01. Are you currently 45 years or older?

1) ☐ Yes、2) ☐ No

02. Have you had sexual health problems at all in the past five years?

1) ☐ Yes、2) ☐ No

03. How would you rate your sexual satisfaction in the past five years?

1) ☐ Very good、2) ☐ Good、3) ☐ Fair、4) Poor

04. How long have you been living in the UK?

1) ☐ Less than 6 months、2) ☐ Greater than 6 months、3) ☐ Not currently living in the UK

**A. DEMOGRAPHICS**

**A1.** What is your current age：______years old?

**A2.** What is your current sex?

1) Male

2) Female

**A1.** What city are you currently living in?

**A2.** What is your highest level of education?

1) ☐ Elementary
2) ☐ Middle school
3) ☐ High school
4) ☐ vocational college
5) ☐ 4-year college
6) ☐ graduate school

**A3.** What is your current marital/relationship status (past 6 months)?



For peer review only - http://bmjopen.bmj.com/site/about/guidelines.xhtml

1) ☐ Single, no significant partner
2) ☐ Currently living with significant partner
3) ☐ Married
4) ☐ Divorced
5) ☐ Widowed
7）☐ Other：________

**A4.** Would you be willing to use sexual health services?
1) Yes
2) No

**A5.** How would you rate your health in general (past 6 months)
1) ☐ excellent、2) ☐ very good、3) ☐ good、4) ☐ fair、5) ☐ poor

## B. Medical history & healthcare utilisation

B1. Have you ever been told by a healthcare professional that you have heart disease, cancer, diabetes, ADHD, a learning disability, asthma, chronic pain, or other chronic disease?
1) ☐ Yes、2) ☐ No [skip to D4]

B2. **If yes**, which one(s)? Check all that apply.
1) ☐ Heart disease、2) ☐ Cancer、3) ☐ Diabetes、4) ☐ ADHD/learning disability、
5) ☐ Asthma、6) ☐ chronic pain、7) ☐ Other [go to D3, otherwise skip to D4]

B3. **If other,** what was it? ______________

B4. In the past 2 years, how often have you visited a doctor?

1) ☐ I have not visited a doctor in the past 2 years、2) ☐ 1-2 times per year、3) ☐ 1-2 times per half-year、4) ☐ 1-2 times per month、5) ☐ 1-2 times per week

B5. In the past 2 years, have you ever avoided going to the doctor even though you were ill or injured?

1) ☐ Yes、2) ☐ No

B6. What type of health insurance do you have?

1) ☐ None、2) ☐ Urban employee、3) ☐ Urban resident、4) ☐ New rural cooperative、5) ☐ Private

B7. Would you be interested in discussing spiritual issues with your healthcare provider?


For peer review only - http://bmjopen.bmj.com/site/about/guidelines.xhtml

1) ☐ Yes、2) ☐ No、3) ☐ Unsure

B8. Do you have a (physical?) disability?

B9. If so, why type of (physical) disability?

B10. Have you ever consulted a healthcare provider for sexual health services (e.g., impotence, STIs, genital pain)?

## C: Sexual behaviours

C1. What is your sexual orientation?
1) ☐ Straight、2) ☐ Gay/homosexual、3) ☐ Bisexual、4) ☐ Other/uncertain

C2. Are you currently in a romantic sexual relationship?
1) ☐ Yes、2) ☐ No

C3. Have you ever had anal/oral/vaginal sex with another person?
1) ☐ Yes、2) ☐ No

C4. How old were you when you first had anal, oral, or vaginal sex? ___________ years old

C5. How many individuals have you ever had anal, oral, or vaginal sex with?
1) ☐ 1、2) ☐ 2-3、3) ☐ 4-5、4) ☐ 6-10、5) ☐ 11-20 、6) ☐ 21-50、7) ☐ >50

C6. Have you ever dated or had relations with anyone who has ever forced you to have sex when you did not want to？
1) ☐ Yes、2) ☐ No

C7. In the past 3 months, have you had sex? (check all that apply)
1) ☐ Yes, vaginal sex、2) ☐ Yes, anal sex、3) ☐ Yes, oral sex、4) ☐ No sex in past 3 months

C8. In the last 3 months, how often did you use a condom or other barrier method during sex?
1) ☐ Every time、2) ☐ Most of the time、3) ☐ Sometimes、4) ☐ Never

C9. Have any of these reasons kept you from getting tested for STI's (sexually transmitted infections)? If there's more than one reason, check all that apply

☐ You have never had sex
☐ It is too expensive
☐ You are afraid of what people might think
☐ You do not think you are at risk



For peer review only - http://bmjopen.bmj.com/site/about/guidelines.xhtml

- ☐ You do not want to know if you have something
- ☐ You do not know where to go to get tested
- ☐ You do not know what is involved in getting tested
- ☐ You do not know what it means to have a positive test
- ☐ Other (Specify): ________________________________

**D: Sexual health services Discrete choice Experiment**

***The following questions will present three sexual health service scenarios (Choice 'A' and 'B'), and a "Do not seek sexual health services" scenario (Choice 'C').*** *Assuming that you have a need for sexual health services [examples or definitions inserted here] , which sexual health doctor consultation scenario would you most prefer? A,B, or C?* **.** ***There are no incorrect answers."***

D1. Please choose the scenario that you prefer the most

| Scenario A | Scenario B | Scenario C |
|---|---|---|
| 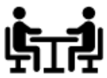<br>Face to face<br>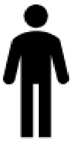<br>Alone with GP<br>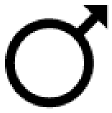<br>Male GP | 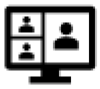<br>Teleconference consultation<br>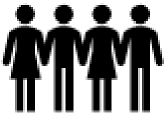<br>Visit GP with family or friends<br>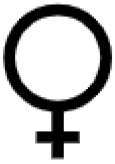<br>Female GP | Do not seek sexual health services |

D2. Please choose the scenario that you prefer the most
…
D3. Please choose the scenario that you prefer the most
…
D4. Please choose the scenario that you prefer the most
…
D5. Please choose the scenario that you prefer the most
…
D6. Please choose the scenario that you prefer the most



For peer review only - http://bmjopen.bmj.com/site/about/guidelines.xhtml

…

D7. Please choose the scenario that you prefer the most

…

D8. Please choose the scenario that you prefer the most

…

For peer review only

BMJ Open

# Preferences for accessing sexual health services among middle-aged and older adults in the UK: a study protocol for a discrete choice experiment using mixed methods

| | |
|---|---|
| Journal: | *BMJ Open* |
| Manuscript ID | bmjopen-2022-066783.R1 |
| Article Type: | Protocol |
| Date Submitted by the Author: | 21-Oct-2022 |
| Complete List of Authors: | Kpokiri, Eneyi; London School of Hygiene and Tropical Medicine Faculty of Infectious and Tropical Diseases, Department of Clinical Research<br>Pan, Stephen W.; Xi'an Jiaotong-Liverpool University, Department of Health and Environmental Sciences<br>Ong, Jason; Monash University; London School of Hygiene & Tropical Medicine<br>Greaves, Emily; London School of Hygiene & Tropical Medicine<br>Khan, Junead; London School of Hygiene & Tropical Medicine<br>Bowen, Sophie; London School of Hygiene and Tropical Medicine Faculty of Infectious and Tropical Diseases, Department of Clinical Research<br>Jannaway, Tracey; Independent Living Alternatives<br>Terris-Prestholt, Fern; London School of Hygiene and Tropical Medicine<br>Tanton, Clare; London School of Hygiene and Tropical Medicine, Global Health & Development<br>Kuper, Hannah; The London School of Hygiene & Tropical Medicine, Clinical Research<br>Shakespeare, Tom; London School of Hygiene and Tropical Medicine Faculty of Infectious and Tropical Diseases<br>Tucker, Joseph; University of North Carolina Project China; London School of Hygiene and Tropical Medicine Faculty of Infectious and Tropical Diseases, Department of Clinical Research<br>Wu, Dan; London School of Hygiene and Tropical Medicine Faculty of Infectious and Tropical Diseases, Department of Clinical Research |
| <b>Primary Subject Heading</b>: | Sexual health |
| Secondary Subject Heading: | Geriatric medicine, Health services research |
| Keywords: | Sexual dysfunction < UROLOGY, SEXUAL MEDICINE, GERIATRIC MEDICINE |
| | |

SCHOLARONE™
Manuscripts

# Preferences for accessing sexual health services among middle-aged and older adults in the UK: a study protocol for a discrete choice experiment using mixed methods

*Eneyi E. Kpokiri[1], *Stephen W. Pan[2], Jason J. Ong[1,3], Emily Greaves[1], Junead Khan[1], Sophie Bowen[1], Tracey Jannaway[4], Fern Terris-Prestholt[5], Clare Tanton[5], Hannah Kuper[1], Tom Shakespeare[1], Joseph D. Tucker[1,6], Dan Wu[1,7]

[1] Department of Clinical Research, London School of Hygiene and Tropical Medicine, London, United Kingdom

[2] Department of Health and Environmental Sciences, Xi'an Jiaotong-Liverpool University, Suzhou, China.

[3] Central Clinical School, Monash University, Melbourne, Australia

[4] Independent Living Alternatives, London, United Kingdom

[5] Faculty of Public Health & Policy, London School of Hygiene & Tropical Medicine, London, United Kingdom

[6] Division of Infectious Diseases, Department of Medicine, University of North Carolina at Chapel Hill, Chapel Hill, NC, United States

[7] Department of Epidemiology, West China School of Public Health, Sichuan University, Chengdu, China

*These authors contributed equally

Corresponding author:

Dan Wu, Department of Epidemiology, West China School of Public Health, Sichuan University, Chengdu, China

Department of Clinical Research, London School of Hygiene and Tropical Medicine, London, United Kingdom

Email: dan.wu@lshtm.ac.uk

For peer review only

## ABSTRACT

**Introduction:** Sexual health is essential for general health and wellbeing. Sexual health services for middle-aged and older adults are not prioritized and optimizing available services for this population is often overlooked. Not much is known about preferences for accessing sexual health services among middle-aged and older people or level of satisfaction with current services. The aim of this study is to explore preferences for seeking sexual health services among middle-aged and older adults in the UK. The study will utilise Discrete Choice Experiments (DCEs) including initial qualitative interviews followed by the survey, which have been used as a tool to explore preferences in various health service delivery.

**Methods and Analysis:** The project will be carried out in two phases. First, we will conduct in-depth semi structured interviews with 20-30 adults (aged 45+), including disabled people, and those from sexual minority groups resident in the UK. Interviews will explore indications, preferences and factors related to accessing sexual health services. Themes and sub-themes emerging from the analysis of the interviews will then be used to design the choice sets and attribute level for the DCEs. For the second phase, for the DCEs, we will design choice sets composed of sexual health service delivery scenarios. The software NGENE will be used to develop the experimental design matrix for the DCE. We will use descriptive statistics to summarize the key sociodemographic characteristics of the study population. Multinomial logit (MNL), latent class (LC) and mixed logit (MXL) models will be explored to assess sexual health service preferences and preference heterogeneity.

**Ethics and Dissemination:** Ethical approval for both parts of this study was granted by the Research and Ethics Committee at the London School of Hygiene and Tropical Medicine, United Kingdom (26134). Findings from this study will be disseminated widely to relevant stakeholders via scheduled meetings, webinars, presentations at conferences and in peer reviewed journal manuscripts.


### Strengths and Limitations of this study

- This is the first DCEs exploring preferences for sexual health services among middle-aged and older adults including disabled people.
- Our DCE is designed based on detailed formative research whereby choice sets are developed from emerging themes from 21 semi-structured interviews



For peer review only - http://bmjopen.bmj.com/site/about/guidelines.xhtml

- While the survey targets mature adults (age 45 and older), we sought to ensure inclusion of those from sexual minority groups and disabled people by recruiting from local community-based organisations. This is to ensure diversity within this population to allow for sub-analysis
- Recruitment will be restricted to only adults aged 45 and older and living in the UK as a result, generalising the findings may be restricted to only the UK or other places with similar settings.

**Background**

Sexual health is an integral part of health, wellbeing, and quality of life. However, many existing sexual health programmes, interventions and research are tailored for youth and younger adults.[1] The sexual health needs of middle-aged and older adults are often neglected, including by health professionals.[2] There are also a host of diverse challenges and barriers that interplay in how and why this age group access sexual health services. Older adults are increasingly experiencing higher rates of co-morbid illnesses further complicating their sexual health and well-being. Up to 9% increase in Sexually Transmitted Infections (STIs) among adults aged 50 and above have been reported. The rate of HIV infections is increasing faster among people aged 50 and above than in the under 40s, consequently, the number of people aged 50 and over living with HIV has now doubled in the last decade.[3,4] Older adults typically turn to family and friends to seek support for health needs and a study assessing sexual health clinic attendance in Britain found that non-attendance was higher among older adults. [5,6] Accordingly, there is suboptimal uptake of sexual health services among middle aged and older adults.[7,8] Within the broader group of "older people", certain groups may be particularly neglected, such as disabled older people, as they face additional barriers to accessing health services.[9] This is an important issue, as almost one in five people in the UK are aged 65+, and 42% of people in this age group report a disability.[10] Disability is defined under the Equality act of 2010 as having a physical or mental impairment that has substantial and long-term negative effect on the ability to do normal daily activities.[11] Despite the growing population of middle-aged and older adults and an increasing body of evidence suggesting sexual health is important to quality of life, there is limited evidence to support preferences and tailor services for specific groups within this population.[12,13]

This study will explore preferences for seeking sexual health services among middle-aged and older adults. This is part of a larger study to inform areas for improved sexual health services for adults aged 45 and above, including disabled ones and from sexual minority



For peer review only - http://bmjopen.bmj.com/site/about/guidelines.xhtml

groups living in the United Kingdom. We have chosen 45 years as our cut off age as menopause, erectile dysfunction and other sexual health issues become more common in that age group. Our study findings will explore issues related to service uptake and identify strategies to increase the uptake of sexual health services among adults aged 45 and above in the UK.

Discrete choice experiments (DCEs) are a quantitative approach to measure strength of preferences. It is an attribute-based measure of utility, based on the assumptions that healthcare interventions, services or policies, can be described by their attributes and that user preferences depend upon the levels of these attributes.[14] DCEs ask participants to make choices from hypothetical alternatives. They are increasingly used in health research to study patient and/or physician preferences.[15] For instance, DCEs have been used to determine preferences for HIV prevention and testing among a range of specific populations and settings to inform strategies for optimising health services.[16-21] DCEs have also been employed to study preferences for social care among older adults.[22] However, to date, no DCE studies have been conducted to determine preferences for sexual health services among older adults. Our study will use DCEs to explore preferences for accessing sexual health services and any heterogeneity that exists among middle-aged and older adults in the UK.

**Overall aim and objectives of this study**

The main aim of this study is to identify strategies to improve access of sexual health services among adults aged 45 and above including sexual minorities and disabled people, in the UK. Specific objectives include:

1) To identify barriers and facilitators for accessing sexual health services among adults aged 45 and above including sexual minorities and disabled people, in the UK using semi-structured interviews

2) To explore key preferences for accessing services for sexual health using a DCE survey

**Methods and Analysis**

**Overview of approach and methods**

The research will be conducted in two phases. First, we will organise semi-structured interviews with adults aged 45 or above to better understand their preferences and the



For peer review only - http://bmjopen.bmj.com/site/about/guidelines.xhtml

importance of sexual health service and message sharing attributes in influencing their use of these services. Interview data will be analysed thematically, and emerging themes will inform which attributes and levels will be included in the choice sets for the DCEs.

**Patient and Public Involvement**

A key part of this study is community engagement as all studying findings will be dependent on response and input from the community members. With the aim of improving access to services, resulting strategies and solutions will be well received and more sustainable with sound community input. Consequently, community representatives have been engaged in the study design through local community-based organisations and reaching out through GP service. These will be continuously engaged in our survey distribution and participant recruitment, data interpretation, reporting, or dissemination plans of our research.

**Phase I- Semi-structured interviews**

DCEs are increasingly being recommended in health research to explore patient preferences.[22] This includes undertaking a conjoint analysis where related characteristics will be identified early on using literature reviews, group discussions or individual interviews.[23] First, we will conduct between 20 to 30 semi-structured individual interviews with adults aged 45 and above and stop when data saturation is reached. This formative qualitative research will identify factors that are considered in the decision-making processes among this population related to sexual health services (e.g., awareness, location, access process, quality of care or qualifications of healthcare providers, cost), identifying barriers and facilitators to service utilisation and sexual health message sharing based on older adults' perceptions of and/or experiences with sexual health services and interaction with sexual health messages. We will also field test the questionnaire using cognitive interviews prior to official start to help us understand how older individuals mentally process and respond to survey questions and DCE choice sets.

Participants will be purposively selected across different locations, age range, ethnic backgrounds, and disability status. Recruitment will be via community-based organisations. We will create and disseminate a simple survey to collect data on profile including age, gender, ethnic background location in the UK, sexual orientation, relationship, and disability status. We will reach out to community-based organisations to disseminate this survey within their members and networks. From the responses received we will then contact participants


For peer review only - http://bmjopen.bmj.com/site/about/guidelines.xhtml

purposively selected to ensure good spread and diversity in the sample recruited for interviews. Interviews will be conducted via telephone calls or teleconference using Zoom, Skype, or Microsoft Teams and facilitated by an experienced qualitative researcher. Teleconference calls may be either audio or video according to participant preferences. Interviews will be conducted using a topic guide (See Appendix I) and we will aim for approximately 30 minutes per interview session. Adjustments will be made as needed to promote the inclusion of disabled persons (e.g., use of sign language interpretation, simple language, inclusion of breaks). Each participant will receive an honorarium worth £30 in appreciation for their participation. A consent form will be provided to participants vie email, which will be signed and returned before the interview commences. Verbal consent will also be obtained right at the start of interview. Interviews will be audio-recorded following participant consent and transcribed verbatim prior to analysis.

**Qualitative data analysis**

We will undertake thematic analysis of the qualitative interview data using NVivo. A codebook will be developed both inductively and deductively to guide analysis. We will employ a framework approach and transcribe all interviews verbatim then commence coding of each transcript. Codes will be aggregated to build up the emerging themes. Outcomes of qualitative data analysis will be the indications and use of sexual health services, preferences and factors affecting their decision making to seek intimate and/or sexual relationships, barriers and facilitators to sexual health service utilisation. This qualitative data generated will be used to inform the DCE survey to be disseminated to participants in phase II. We will also disseminate the data as presentations in scientific conferences and publications in open access journals (See Appendix II for some preliminary results).

**Data Management**

All data (qualitative and quantitative) collected during the course of the research will be kept strictly confidential and will not be shared with anyone outside the research team. The identity of participants will be known by the researchers who will assign participant identity numbers. Responses will be de-identified prior to analysis, and results will be reported at an aggregate level, so that participants cannot be identified.


For peer review only - http://bmjopen.bmj.com/site/about/guidelines.xhtml

## Phase II- Discrete Choice Experiment survey

Second, we will use the qualitative information from Phase I to inform what attributes (characteristics) and levels (choices) will be included in the unlabeled choice set, which will be administered in an online survey to a sample of older adults to identify their preferences for accessing sexual health services. We will design choice sets composed of hypothetical sexual health service delivery scenarios. Similarly, as stated for the interview, we will employ same strategy to recruit disabled people and people from sexual minority groups for the survey to allow sub-analyses of the results. We will aim to have disabled people and sexual minorities make up 10% each out of the total sample size.

## Developing the DCE design

*Pictorial representation of attribute levels and DCE choice set size*

A ranking exercise will be organized with some of the interview participants to align and finalise the attributes and levels that will be included in the final choice sets (Appendix III). After attributes and levels have been finalised, we will generate visual graphics to represent each attribute level in the DCE survey (See Appendix IV for a sample). To ensure that the visual graphics effectively represent the intended meaning of each attribute level (i.e., construct validity), we will conduct qualitative think-aloud, pilot exercises with up to 10 individuals from the target population, including disabled people and those from sexual minority groups and participants will provide feedback about how they interpret and feel about the graphics.[24] This will be an iterative process with the draft pictorial concepts being tested until they are appropriately representing the intended concept. DCE surveys tend to present between 4 and 18 choice sets. During these interviews, we will also explore the optimal number of choice sets to present to each respondent to manage potential cognitive fatigue. Similar to the phase 1 interviews, participants will be purposively selected across different locations, age range, ethnic backgrounds and disability status. Again, reasonable accommodation will be made to promote inclusion of disabled persons, although people with severe cognitive/intellectual impairment will not be included in this step. Recruitment will be through collaborations with community-based organisations with which our research team already have existing research collaborations with.

*Experimental design*

For peer review only

After visual graphics of the attribute levels have been finalised, we will use the software NGENE to develop the experimental design matrix for the DCE pilot.[25] For the pilot test, no assumptions about the parameter estimates will be made. We will then specify the appropriate number of attributes, levels, alternatives, blocks, and choice sets, and NGENE will iteratively generate design matrices of increasing statistical efficiency. We will use the design with the lowest D-error generated by NGENE. An example of a choice set and DCE design is presented in Figure 1. Graphics will have alt-text enabled so that they can be accessed by people with visual impairments.

*Blocking and randomisation of choice set ordering*

Depending on the total number of choice sets in the DCE design, choice sets may be grouped into blocks. Respondents will then be randomly assigned to one of the blocks and asked to complete all choice sets in their block. We anticipate using between 4-10 blocks, with each block containing 4-10 choice sets. To guard against potential ordering effects, the order of the choice sets within each block will be randomised for each participant.

*Pilot testing*

We intend to use convenience sampling to recruit up to 30 participants for the pilot test. Participants in the pilot test will receive a £20 gift card for completing the survey. Conducting the pilot test will serve two purposes: (1) identify potential problems with the DCE and survey in general (e.g., cognitive fatigue or incoherent questions or lack of accessibility) and (2) generate priors (i.e., parameter estimates) that will be used to develop the design of the final full-scale DCE. We will analyse the pilot data with multinomial logit models (MNL), given the limited sample size. Priors from the pilot test will be input into NGENE to finalise and enhance the efficiency of the full-scale DCE design.

*Full-scale DCE survey*

Community-based organizations, local clinics, GP practices and older people care homes will be contacted to help distribute the online survey. Recruitment will be carried out purposively from urban and suburban areas, evenly spread across the middle age and older adults age groups. We will create formats for completing the DCE surveys both online and offline.

**Eligibility criteria:**



For peer review only - http://bmjopen.bmj.com/site/about/guidelines.xhtml

We will recruit persons who meet all of the following criteria:

1) Aged 45 years or older

2) Had the experience of accessing sexual reproductive health service', prior to this study

3) Resident in the UK for at least six months

4) Willing to consent to participation.

With consent, participants of the DCE will be entered into a draw where up to 20 participants will be randomly selected to receive a £100 gift card.

*Quality assessment*

It is possible that some **participants** known as "speeders" may try to complete the DCE survey as soon as possible without seriously considering the survey questions. "Speeders" will be identified as those who complete the survey at least 50% faster than the median time for survey completion among all valid survey respondents. We will then conduct a sensitivity analysis and determine whether or not the removal of identified speeders significantly alters the results.

We will also assess the possibility of order effect bias by introducing a dummy variable for the order of the choice set. We will test for left and right bias by including a constant for the left choice and examining the extent to which the left position of the alternatives in the choice sets influences the likelihood of being chosen. Comparable probabilities of the left and centre alternatives being selected would suggest that the absence of bias.

**Sample size calculation**

Given the absence of any DCE priors for sexual health preferences of older adults in the UK, we used the following parametric-based DCE sample size calculation formula

$$n \geq \frac{(1-p)}{rpa^2} \times \left[ (\Phi^{-1}\left(1-\frac{\alpha}{2}\right) \right]^2$$

**Equation 1: Parametric DCE sample size formula[26]**

Whereby $n$ is the minimum sample size needed to detect $p$, the assumed true population probability of selecting uptake of sexual health services over "opt-out", and whereby $r$ is the


For peer review only - http://bmjopen.bmj.com/site/about/guidelines.xhtml

number of choice sets that each individual completes, $a$ is the level of precision around the assumed population probability, $\Phi^{-1}$ is the inverse cumulative normal distribution function, and $\alpha$ is the significance level. Conservatively assuming that the true population probability of sexual health service uptake is 0.5 and assuming that each participant will complete eight choice sets, we estimate that a sample of 191 participants will be needed to generate a point estimate ± 0.05 of the true population probabilities and with 95% confidence. However, to maintain power for two-group subgroup analyses, we will aim to recruit a total **240** participants including those from sexual minorities groups and disabled people.

**Statistical analysis**

We will use descriptive statistics to summarize the key sociodemographic characteristics of the study population. We will assess sexual health service preferences and preference heterogeneity using multinomial logit (MNL), latent class (LC) and mixed logit (MXL) models. While the MNL is the starting point for most discrete choice models, it suffers from some unrealistic assumptions, particularly around unobserved heterogeneity. The MXL model can accommodate unobserved heterogeneity. The MXL analysis will be conducted with1000 Halton draws and will assume normally distributed preference heterogeneity for each attribute level. We will assess for unobserved heterogeneity by examining the statistical significance of the standard deviations of the random parameters in the MXL model. We will calculate the relative importance and predicted probabilities of opt-out for select sexual health service scenarios. We will assess how sexual health service and message sharing preferences could vary among those willing and unwilling to use sexual health services by using interaction terms for sociodemographic characteristics.

We will perform latent class analyses to elucidate if there are unobserved groups of people with similar preferences for use of sexual health service.[27] First, we will conduct a latent class conditional logit model that includes individual characteristics and attribute level preferences.[27] Selection of individual characteristics will be determined by expert opinion and findings from the formative qualitative interviews. Second, we will determine the optimal number of subgroups by comparing the BIC values of 2, 3, 4, 5, 6, and 7-class models and each models' interpretability and class size distribution.[27] Third, we will use descriptive statistics to characterise each class based on their members' preferences and characteristics. All analyses will use effects coding and be weighted to reflect the population of middle-aged and older adults in the UK.

For peer review only

*Attribute relative importance and probability of opt-out*

For each of the sex-class combinations, the relative importance of each attribute will be calculated by dividing the range of the parameter estimates of a given attribute by the summation of the ranges of all attributes.[28]

We will further illustrate potential preference heterogeneities by using equation 2 to calculate the predicted probabilities of selecting "opt-out" over select sexual health service scenarios for each sex-class combination. Specifically, the predicted probability of a participant choosing alternative *i* over alternative *j* can be estimated by dividing the exponentiated summation of coefficients for scenario *i* by the exponentiated summation of coefficients for scenario *j*.

$$\mathrm{Pi} = \frac{e^{\beta x_i}}{\sum e^{\beta x_j}} \quad \mathrm{Pi} = \frac{e^{\beta x_i}}{\sum e^{\beta x_j}}$$

**Equation 2: Predicted probability of selecting alternative *i* within a two-alternative choice set**

**Impact**

This study will improve our understanding of older people's preferences and factors important for sexual health services. Our data will be useful to develop sexual health services and programs tailored for older people. By engaging older adults, this study will raise awareness for the need to access sexual health services irrespective of age and also ultimately identify strategies co-created by the end-users to increase service uptake.

**Feasibility**

Our project is feasible as the team has expertise from multiple disciplines to examine sexual health among older adults with unique strengths in DCEs, disability studies, and sexual health research.

**Funding**

The study is supported by Economic and Social Research Council, UK Research and Innovation (UKRI) [grant number: ES/T014547/1].

**Ethical approval**

For peer review only



For peer review only - http://bmjopen.bmj.com/site/about/guidelines.xhtml

Ethical approval was obtained from the Research and Ethics Committee at the London School of Hygiene and Tropical Medicine, United Kingdom (26134)

**Competing interests**

None.

**Contributors Statement**

DW and JDT conceived the idea. DW oversees the study. EEK and SWP wrote the first draft of the protocol paper. EEK, EG and SB conducted the interviews, developed attributes and levels, and JK helped refine the DCE survey design. JJO, SWP, FTP and JDT provided additional advice on the discrete choice experiment design and will carry out the experiment design. TJ, CT, HK, TS and JDT provided expertise advice on improving inclusiveness and appropriateness of the protocol. All authors reviewed, provided constructive comments on improving the design and the manuscript. All authors have seen and approve the final version of this manuscript.

**References:**

1. Ezhova I, Savidge L, Bonnett C, Cassidy J, Okwuokei A, Dickinson T. Barriers to older adults seeking sexual health advice and treatment: A scoping review. *International journal of nursing studies*. 2020;107:103566.
2. McGrath M, Lynch E. Occupational therapists' perspectives on addressing sexual concerns of older adults in the context of rehabilitation. *Disability and rehabilitation*. 2014;36(8):651-657.
3. UK N. Report looks at the Health of Baby Boomers. Accessed 17 October, 2022. www.nhs.uk/news/older-people/report-looks-at-the-health-of-the-baby-boomer-generation/
4. Aging BRIo. Sexually Transmitted Diseases in Older Adults. Accessed 17 October, 2022. www.benrose.org/Resources/article-stds-older-adults.cfm
5. Brennan-Ing M, Seidel L, London AS, Cahill S, Karpiak SE. Service utilization among older adults with HIV: The joint association of sexual identity and gender. *Community-Based Research on LGBT Aging*. Routledge; 2017:166-196.
6. Tanton C, Geary RS, Clifton S, et al. Sexual health clinic attendance and non-attendance in Britain: findings from the third National Survey of Sexual Attitudes and Lifestyles (Natsal-3). *Sexually transmitted infections*. 2018;94(4):268-276.
7. Gott M, Hinchliff S, Galena E. General practitioner attitudes to discussing sexual health issues with older people. *Social science & medicine*. 2004;58(11):2093-2103.
8. Beaudreau SA, Van Moorleghem K, Dodd SM, Liou-Johnson V, Suresh M, Gould CE. Satisfaction with a vacuum constriction device for erectile dysfunction among middle-aged and older veterans. *Clinical Gerontologist*. 2021;44(3):307-315.


For peer review only - http://bmjopen.bmj.com/site/about/guidelines.xhtml

9. Organization WH. *Summary: World report on disability 2011*. 2011. 6099570705.
10. GOV.UK. Family Resources Survey: financial year 2020 to 2021. Accessed 14 June, 2022. https://www.gov.uk/government/statistics/family-resources-survey-financial-year-2020-to-2021/family-resources-survey-financial-year-2020-to-2021#disability-1
11. GOV.UK. Equality Act 2010. GOV.UK. Accessed 02 March 2022, 2022. https://www.gov.uk/definition-of-disability-under-equality-act-2010#:~:text=You're%20disabled%20under%20the,to%20do%20normal%20daily%20activities.
12. Lindau ST, Schumm LP, Laumann EO, Levinson W, O'Muircheartaigh CA, Waite LJ. A study of sexuality and health among older adults in the United States. *New England Journal of Medicine*. 2007;357(8):762-774.
13. Hinchliff S, Tetley J, Lee D, Nazroo J. Older adults' experiences of sexual difficulties: qualitative findings from the English Longitudinal Study on Ageing (ELSA). *The Journal of Sex Research*. 2018;55(2):152-163.
14. Ryan M, Gerard K. Using discrete choice experiments to value health care programmes: current practice and future research reflections. *Applied health economics and health policy*. 2003;2(1):55-64.
15. Quaife M, Terris-Prestholt F, Di Tanna GL, Vickerman P. How well do discrete choice experiments predict health choices? A systematic review and meta-analysis of external validity. *The European journal of health economics*. 2018;19(8):1053-1066.
16. Strauss M, George G, Lansdell E, et al. HIV testing preferences among long distance truck drivers in Kenya: a discrete choice experiment. *AIDS care*. 2018;30(1):72-80.
17. Ong JJ, Nwaozuru U, Obiezu-Umeh C, et al. Designing HIV Testing and Self-Testing Services for Young People in Nigeria: A Discrete Choice Experiment. *The Patient-Patient-Centered Outcomes Research*. 2021:1-12.
18. Schaffer EM, Gonzalez JM, Wheeler SB, Kwarisiima D, Chamie G, Thirumurthy H. Promoting HIV testing by men: a discrete choice experiment to elicit preferences and predict uptake of community-based testing in Uganda. *Applied health economics and health policy*. 2020:1-20.
19. Ong JJ, Nwaozuru U, Obiezu-Umeh C, et al. Designing HIV Testing and Self-Testing Services for Young People in Nigeria: A Discrete Choice Experiment. *The Patient-Patient-Centered Outcomes Research*. 2021;14(6):815-826.
20. Pan SW, Durvasula M, Ong JJ, et al. No place like home? Disentangling preferences for HIV testing locations and services among men who have sex with men in China. *AIDS and Behavior*. 2019;23(4):847-859.
21. Terris-Prestholt F, Mulatu A, Quaife M, et al. Using choice experiments to improve equity in access to socially marketed HIV prevention products. *Journal of choice modelling*. 2021;41:100319.
22. Ryan M, Netten A, Skåtun D, Smith P. Using discrete choice experiments to estimate a preference-based measure of outcome—an application to social care for older people. *Journal of health economics*. 2006;25(5):927-944.
23. Ryan M, Farrar S. Using conjoint analysis to elicit preferences for health care. *Bmj*. 2000;320(7248):1530-1533.
24. Aday LA, Cornelius LJ. *Designing and conducting health surveys: a comprehensive guide*. John Wiley & Sons; 2006.
25. Metrics C. NGENE 1.1. 2 User Manual and Reference Guide: the cutting edge in experimental design. *USA: Choice Metrics*. 2014;

26. de Bekker-Grob EW, Donkers B, Jonker MF, Stolk EA. Sample size requirements for discrete-choice experiments in healthcare: a practical guide. *The Patient-Patient-Centered Outcomes Research*. 2015;8(5):373-384.
27. Zhou M, Thayer WM, Bridges JF. Using latent class analysis to model preference heterogeneity in health: a systematic review. *Pharmacoeconomics*. 2018;36(2):175-187.
28. Hauber AB, González JM, Groothuis-Oudshoorn CG, et al. Statistical methods for the analysis of discrete choice experiments: a report of the ISPOR conjoint analysis good research practices task force. *Value in health*. 2016;19(4):300-315.

For peer review only

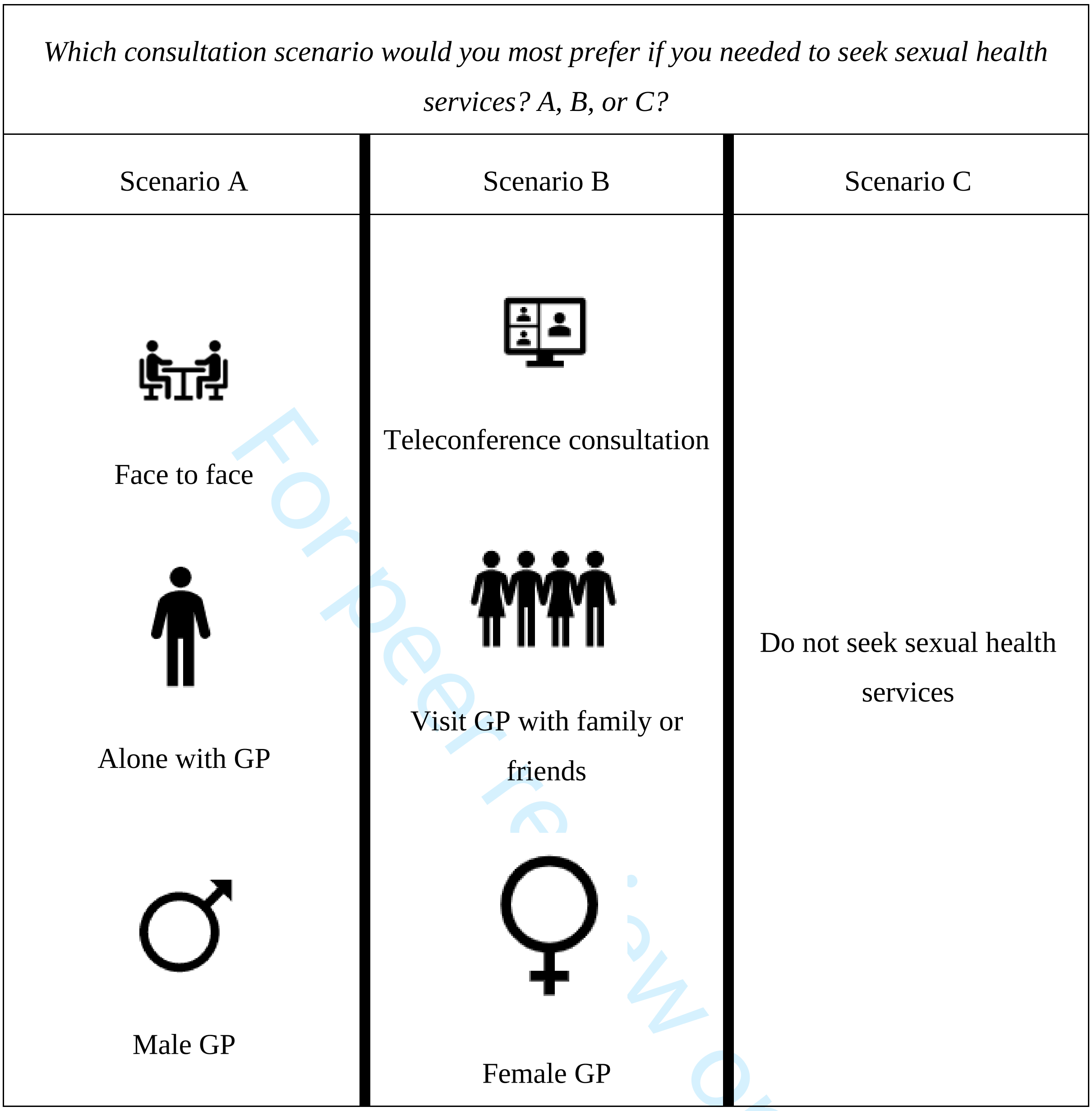


**Figure 1: Fictitious example of a choice set**

For peer review only

## Appendix I

## Sexual health in middle age and older adults in the UK
## Interview Topic Guide

Date of interview:

Respondent ID:

**Social Demographics**

Sex:

Age:

Ethnic group:

Local County:

Do you consider yourself to have a disability?

**Introduction (to be read by interviewer just before commencing the interview):**

Thank you for accepting to take part in this interview. I would just like to reiterate that everything you say in the interview is confidential. All data collected will be anonymized. The interview itself will be open ended and the questions themselves are usually broad. There aren't any right or wrong answers. I'm simply interested in your experience and your views about sexual health services. Sexual health is fundamental to the overall wellbeing of many individuals. Is there anything you'd like to ask me?

**Part I: Sexual health services**

1. Have you used sexual health services in the last (6-12months)?
2. If no, any reasons?
3. If yes, were you satisfied with the service received?
4. Overall, how would you rate sexual health services you received?
   <Very satisfied, Satisfied, Neutral, Dissatisfied, Very dissatisfied>
5. Do you feel sexual health services available to older adults are adequate?
6. Do you talk to your GP about sexual health concerns?
7. Does your GP bring up sexual health related discissions with you?
8. Do you feel comfortable posting or commenting on sexual health topics on social media?



For peer review only - http://bmjopen.bmj.com/site/about/guidelines.xhtml

**Part II: Decision making process**

1. What factors impact on your decision to seek sexual health services?
2. What would you access sexual health services for? General information and advice? Sexual function? STI testing?
3. How would you prefer to access sexual health services? Face to face or via telephone consultation? Or just online information?
4. For sexual health issues would you prefer to see a doctor that is:
    a. Same sex as you?
    b. Same ethnic background as you? Or speaks same language as you?
    c. A junior doctor or Very senior/ advanced doctor
    d. Have a friend, relation, partner go with you?
5. Where would you rather go for sexual health services? GP, Hospital, Pharmacy, Online, Older people groups/organisations medical outreach?

**Part III: Barriers and facilitators**

1. What factors prevent you from accessing sexual health services?
2. How would you prefer to access sexual health services?
3. What factors would make sexual health services easy and comfortable for you?
4. Would you be willing to participate in an open call and share messages about sexual health? With whom might you consider or not consider sharing the messages with? If so, what possible means of sharing would you prefer?

For peer review only

## Appendix II: Preliminary qualitative results

So far, we have recruited and conducted 9 participants for the qualitative interviews as part of the first phase for the DCE project. Below are some preliminary results.

***Socio-demographic characteristics of participants***

There was a fair balance in gender with 4 males and 5 female respondents. Respondents were aged between 45 and 73 years with an average age of 58 years. All respondents were heterosexuals with more than half in a married /civil relationship (n=5). Few others were living with partner (n=2) and either single (n=1) or divorced (n=1). Respondents were either white-British (n=4) or black/African/British/Caribbean (n=5). Only one participant reported living with a disability and two-thirds of respondents confirmed using sexual health services within the last one year. See Table 1 below:

**Table 1: Socio-demographics of participants**

| **Characteristics** (n = 9) | **n (%)** |
|---|---|
| **Gender** | |
| Male | 4 (44) |
| Female | 5 (56) |
| **Age** (years) | |
| 45-50 | 3 (66) |
| 51-60 | 2 (22) |
| 61-70 | 3(33) |
| > 70 | 1 (11) |
| **Relationship status** | |
| Married/civil relationship | 5 (56) |
| Single | 1(11) |
| Living with partner | 2 (22) |
| Divorced | 1 (11) |
| **Sexual orientation** | |
| Straight heterosexual | 9 (100) |
| Homosexual gay/lesbian | 0 (0) |
| Bi-sexual | 0 (0) |
| **Ethnicity** | |
| White-British | 4 (44) |
| Black/British/Afro/Caribbean | 5 (56) |
| **Disability** | |
| Yes | 8 (89) |
| No | 1 (11) |
| **Used SH Service in last year?** | |
| Yes | 6 (67) |
| No | 3 (33) |

For peer review only



For peer review only - http://bmjopen.bmj.com/site/about/guidelines.xhtml

*Preferences for accessing sexual health services and sharing messages*

Participants that had used sexual health services were generally satisfied with services received. Most persons would rather not speak to their GP about their sexual health concerns unless it was absolutely necessary. Community sexual health clinics are preferred over GP's. Many older adults have sexual health needs related to decline sexual function and will not share sexual health related messages openly but are willing to send individual messages privately. Clear information about availability of sexual health services is needed.

**Table 2: Preferences for accessing sexual health services and sharing messages**

| Questions | Responses | Themes |
|---|---|---|
| Do you talk to your GP about sexual health concerns | No/ if there is a need to | Most persons do not feel comfortable talking to their regular GP about their sexual health needs |
| Would you feel comfortable sharing messages relating to sexual health topics on social media? | Majority – No<br>Can send message privately to family friend if needed | Respondents will not share sexual health related messages openly but are willing to send individual messages privately if there is a need |
| How would you prefer to access sexual health services? | Mixed responses- Face to face or online | Depending on the severity of the sexual health problem either face to face or an initial telephone/online consultation is preferred |
| What would you access sexual health services for? | Sexual function<br>STI testing<br>Contraception<br>General info/advice<br>Pre-menopausal concerns<br>Assisted reproduction | Many older adults have sexual health needs related to decline sexual function<br><br>There is need for clearer information on what services are available and how to access them |
| Where would you rather go for sexual health services? | Community NHS clinics, Not GP<br>Anonymous NHS staff<br>Online? | Community sexual health clinics are preferred over GP's. |
| What factors would you consider when accessing sexual health services? | Convenience, quality of service (professional), anonymity, discretion, | With sexual health quality and professional services is always key. A platform that is easy and convenient is also important |



For peer review only - http://bmjopen.bmj.com/site/about/guidelines.xhtml

## Appendix III: Draft Attributes and levels for ranking

| Attributes | Levels |
|---|---|
| **Service Type** | |
| *Service provider for consultation* | • GP clinic<br>• Sexual health clinic<br>• Pharmacy<br>• Online Platform |
| *Mode of delivery* | • Face to face<br>• Telephone call<br>• Video conference<br>• Online chat function |
| *Provider initiates discussion of sexual health* | • Yes<br>• No |
| *Anonymity* | • Name-based<br>• Partially anonymous<br>• Totally anonymous |
| **Provider Characteristics** | |
| *Gender ethnicity and age of health personnel* | • Same gender, age range and ethnicity<br>• Different gender age range and ethnicity |
| *Seniority of health personnel* | • Fairly recently qualified provider<br>• Older, more established provider |
| *Relationship with health personnel* | • Familiar provider/ existing relationship<br>• New provider |
| *Personnel with intersectional training (incl language on gender and sexual minorities, disabilities etc)* | • With intersectional training<br>• Without intersectional training |
| **Service Experience** | |
| *Who attends consultation with you* | • Family, friend, or personal care aide<br>• Medical assistant<br>• No one |
| *Provider privacy policy* | • Reassures patient of privacy<br>• No specific mention of privacy |
| *Accessibility* | • Accessible buildings and disability-friendly equipment<br>• Older, not updated accessibility accommodations |

For peer review only

## Appendix IV: Sample DCE survey

**Participant ID:**

Please do not leave any blank questions. There are no wrong answers.

**ELIGIBILITY**

01. Are you currently 45 years or older?

1) ☐ Yes、2) ☐ No

02. Have you had sexual health problems at all in the past five years?

1) ☐ Yes、2) ☐ No

03. How would you rate your sexual satisfaction in the past five years?

1) ☐ Very good、2) ☐ Good、3) ☐ Fair、4) Poor

04. How long have you been living in the UK?

1) ☐ Less than 6 months、2) ☐ Greater than 6 months、3) ☐ Not currently living in the UK

**A. DEMOGRAPHICS**

**A1.** What is your current age：______years old?

**A2.** What is your current sex?

1) Male

2) Female

**A1.** What city are you currently living in?

**A2.** What is your highest level of education?

1) ☐ Elementary
2) ☐ Middle school
3) ☐ High school
4) ☐ vocational college
5) ☐ 4-year college
6) ☐ graduate school

**A3.** What is your current marital/relationship status (past 6 months)?



For peer review only - http://bmjopen.bmj.com/site/about/guidelines.xhtml

1) ☐ Single, no significant partner
2) ☐ Currently living with significant partner
3) ☐ Married
4) ☐ Divorced
5) ☐ Widowed
7）☐ Other：________

**A4.** Would you be willing to use sexual health services?
1) Yes
2) No

**A5.** How would you rate your health in general (past 6 months)
1) ☐ excellent、2) ☐ very good、3) ☐ good、4) ☐ fair、5) ☐ poor

**B. Medical history & healthcare utilisation**

B1. Have you ever been told by a healthcare professional that you have heart disease, cancer, diabetes, ADHD, a learning disability, asthma, chronic pain, or other chronic disease?
1) ☐ Yes、2) ☐ No [skip to D4]

B2. **If yes**, which one(s)? Check all that apply.
1) ☐ Heart disease、2) ☐ Cancer、3) ☐ Diabetes、4) ☐ ADHD/learning disability、
5) ☐ Asthma、6) ☐ chronic pain、7) ☐ Other [go to D3, otherwise skip to D4]

B3. **If other,** what was it? ______________

B4. In the past 2 years, how often have you visited a doctor?

1) ☐ I have not visited a doctor in the past 2 years、2) ☐ 1-2 times per year、3) ☐ 1-2 times per half-year、4) ☐ 1-2 times per month、5) ☐ 1-2 times per week

B5. In the past 2 years, have you ever avoided going to the doctor even though you were ill or injured?

1) ☐ Yes、2) ☐ No

B6. What type of health insurance do you have?

1) ☐ None、2) ☐ Urban employee、3) ☐ Urban resident、4) ☐ New rural cooperative、5) ☐ Private

B7. Would you be interested in discussing spiritual issues with your healthcare provider?


For peer review only - http://bmjopen.bmj.com/site/about/guidelines.xhtml

1) ☐ Yes、2) ☐ No、3) ☐ Unsure

B8. Do you have a (physical?) disability?

B9. If so, why type of (physical) disability?

B10. Have you ever consulted a healthcare provider for sexual health services (e.g., impotence, STIs, genital pain)?

## C: Sexual behaviours

C1. What is your sexual orientation?
1) ☐ Straight、2) ☐ Gay/homosexual、3) ☐ Bisexual、4) ☐ Other/uncertain

C2. Are you currently in a romantic sexual relationship?
1) ☐ Yes、2) ☐ No

C3. Have you ever had anal/oral/vaginal sex with another person?
1) ☐ Yes、2) ☐ No

C4. How old were you when you first had anal, oral, or vaginal sex? __________ years old

C5. How many individuals have you ever had anal, oral, or vaginal sex with?
1) ☐ 1、2) ☐ 2-3、3) ☐ 4-5、4) ☐ 6-10、5) ☐ 11-20 、6) ☐ 21-50、7) ☐ >50

C6. Have you ever dated or had relations with anyone who has ever forced you to have sex when you did not want to？
1) ☐ Yes、2) ☐ No

C7. In the past 3 months, have you had sex? (check all that apply)
1) ☐ Yes, vaginal sex、2) ☐ Yes, anal sex、3) ☐ Yes, oral sex、4) ☐ No sex in past 3 months

C8. In the last 3 months, how often did you use a condom or other barrier method during sex?
1) ☐ Every time、2) ☐ Most of the time、3) ☐ Sometimes、4) ☐ Never

C9. Have any of these reasons kept you from getting tested for STI's (sexually transmitted infections)? If there's more than one reason, check all that apply
☐ You have never had sex
☐ It is too expensive
☐ You are afraid of what people might think
☐ You do not think you are at risk

For peer review only

- ☐ You do not want to know if you have something
- ☐ You do not know where to go to get tested
- ☐ You do not know what is involved in getting tested
- ☐ You do not know what it means to have a positive test
- ☐ Other (Specify): ________________________________

**D: Sexual health services Discrete choice Experiment**

***The following questions will present three sexual health service scenarios (Choice 'A' and 'B'), and a "Do not seek sexual health services" scenario (Choice 'C').*** *Assuming that you have a need for sexual health services [examples or definitions inserted here] , which sexual health doctor consultation scenario would you most prefer? A,B, or C?* ***. There are no incorrect answers."***

D1. Please choose the scenario that you prefer the most

| Scenario A | Scenario B | Scenario C |
|---|---|---|
| 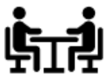 Face to face 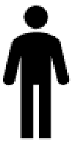 Alone with GP 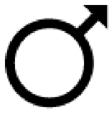 Male GP | 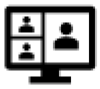 Teleconference consultation 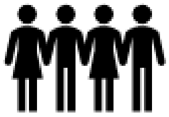 Visit GP with family or friends 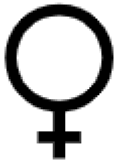 Female GP | Do not seek sexual health services |

D2. Please choose the scenario that you prefer the most
…
D3. Please choose the scenario that you prefer the most
…
D4. Please choose the scenario that you prefer the most
…
D5. Please choose the scenario that you prefer the most
…
D6. Please choose the scenario that you prefer the most

For peer review only

…

D7. Please choose the scenario that you prefer the most

…

D8. Please choose the scenario that you prefer the most

…

For peer review only

# Preferences for accessing sexual health services among middle-aged and older adults in the United Kingdom: a study protocol for a discrete choice experiment using mixed methods

| | |
|---|---|
| Journal: | *BMJ Open* |
| Manuscript ID | bmjopen-2022-066783.R2 |
| Article Type: | Protocol |
| Date Submitted by the Author: | 05-Jan-2023 |
| Complete List of Authors: | Kpokiri, Eneyi; London School of Hygiene and Tropical Medicine Faculty of Infectious and Tropical Diseases, Department of Clinical Research<br>Pan, Stephen W.; Xi'an Jiaotong-Liverpool University, Department of Health and Environmental Sciences<br>Ong, Jason; Monash University; London School of Hygiene & Tropical Medicine<br>Greaves, Emily; London School of Hygiene & Tropical Medicine<br>Khan, Junead; London School of Hygiene & Tropical Medicine<br>Bowen, Sophie; London School of Hygiene and Tropical Medicine Faculty of Infectious and Tropical Diseases, Department of Clinical Research<br>Jannaway, Tracey; Independent Living Alternatives<br>Terris-Prestholt, Fern; London School of Hygiene and Tropical Medicine<br>Tanton, Clare; London School of Hygiene and Tropical Medicine, Global Health & Development<br>Kuper, Hannah; The London School of Hygiene & Tropical Medicine, Clinical Research<br>Shakespeare, Tom; London School of Hygiene and Tropical Medicine Faculty of Infectious and Tropical Diseases<br>Tucker, Joseph; University of North Carolina Project China; London School of Hygiene and Tropical Medicine Faculty of Infectious and Tropical Diseases, Department of Clinical Research<br>Wu, Dan; London School of Hygiene and Tropical Medicine Faculty of Infectious and Tropical Diseases, Department of Clinical Research |
| <b>Primary Subject Heading</b>: | Sexual health |
| Secondary Subject Heading: | Geriatric medicine, Health services research |
| Keywords: | Sexual dysfunction < UROLOGY, SEXUAL MEDICINE, GERIATRIC MEDICINE |
| | |

SCHOLARONE™
Manuscripts

# Preferences for accessing sexual health services among middle-aged and older adults in the United Kingdom: a study protocol for a discrete choice experiment using mixed methods

*Eneyi E. Kpokiri[1], *Stephen W. Pan[2], Jason J. Ong[1,3], Emily Greaves[1], Junead Khan[1], Sophie Bowen[1], Tracey Jannaway[4], Fern Terris-Prestholt[5], Clare Tanton[5], Hannah Kuper[1], Tom Shakespeare[1], Joseph D. Tucker[1,6], Dan Wu[1,7]

[1] Department of Clinical Research, London School of Hygiene and Tropical Medicine, London, United Kingdom

[2] Department of Health and Environmental Sciences, Xi'an Jiaotong-Liverpool University, Suzhou, China.

[3] Central Clinical School, Monash University, Melbourne, Australia

[4] Independent Living Alternatives, London, United Kingdom

[5] Faculty of Public Health & Policy, London School of Hygiene & Tropical Medicine, London, United Kingdom

[6] Division of Infectious Diseases, Department of Medicine, University of North Carolina at Chapel Hill, Chapel Hill, NC, United States

[7] Department of Epidemiology, West China School of Public Health, Sichuan University, Chengdu, China

*These authors contributed equally

Corresponding author:

Dan Wu, Department of Epidemiology, West China School of Public Health, Sichuan University, Chengdu, China

Department of Clinical Research, London School of Hygiene and Tropical Medicine, London, United Kingdom

Email: dan.wu@lshtm.ac.uk



For peer review only

## ABSTRACT

**Introduction:** Sexual health is essential for general health and wellbeing. Sexual health services for middle-aged and older adults are not prioritized and optimizing available services for this population is often overlooked. Not much is known about preferences for accessing sexual health services among middle-aged and older people or level of satisfaction with current services. The aim of this study is to explore preferences for seeking sexual health services among middle-aged and older adults in the United Kingdom. The study will utilise Discrete Choice Experiments (DCEs) including initial qualitative interviews followed by the survey, which have been used as a tool to explore preferences in various health service delivery.

**Methods and Analysis:** The project will be carried out in two phases. First, we will conduct in-depth semi structured interviews with 20-30 adults (aged 45+), including disabled people, and those from sexual minority groups resident in the UK. Interviews will explore indications, preferences and factors related to accessing sexual health services. Themes and sub-themes emerging from the analysis of the interviews will then be used to design the choice sets and attribute level for the DCEs. For the second phase, for the DCEs, we will design choice sets composed of sexual health service delivery scenarios. The software NGENE will be used to develop the experimental design matrix for the DCE. We will use descriptive statistics to summarize the key sociodemographic characteristics of the study population. Multinomial logit, latent class and mixed logit models will be explored to assess sexual health service preferences and preference heterogeneity.

**Ethics and Dissemination:** Ethical approval for both parts of this study was granted by the Research and Ethics Committee at the London School of Hygiene and Tropical Medicine. Findings from this study will be disseminated widely to relevant stakeholders via scheduled meetings, webinars, presentations and journal publications.


**Strengths and Limitations of this study**

- This is the first DCEs exploring preferences for sexual health services among middle-aged and older adults including disabled people.
- Our DCE is designed based on detailed formative research whereby choice sets are developed from emerging themes from 21 semi-structured interviews

For peer review only

- While the survey targets mature adults (age 45 and older), we sought to ensure inclusion of those from sexual minority groups and disabled people by recruiting from local community-based organisations. This is to ensure diversity within this population to allow for sub-analysis
- Recruitment will be restricted to only adults aged 45 and older and living in the UK as a result, generalising the findings may be restricted to only the UK or other places with similar settings.

## Introduction

Sexual health is an integral part of health, wellbeing, and quality of life. However, many existing sexual health programmes, interventions and research are tailored for youth and younger adults.[1] The sexual health needs of middle-aged and older adults are often neglected, including by health professionals.[2] There are also a host of diverse challenges and barriers that interplay in how and why this age group access sexual health services. Older adults are increasingly experiencing higher rates of co-morbid illnesses further complicating their sexual health and well-being. Up to 9% increase in Sexually Transmitted Infections (STIs) among adults aged 50 and above have been reported. The rate of HIV infections is increasing faster among people aged 50 and above than in the under 40s, consequently, the number of people aged 50 and over living with HIV has now doubled in the last decade.[3,4] Older adults typically turn to family and friends to seek support for health needs and a study assessing sexual health clinic attendance in Britain found that non-attendance was higher among older adults. [5,6] Accordingly, there is suboptimal uptake of sexual health services among middle aged and older adults.[7,8] Within the broader group of "older people", certain groups may be particularly neglected, such as disabled older people, as they face additional barriers to accessing health services.[9] This is an important issue, as almost one in five people in the UK are aged 65+, and 42% of people in this age group report a disability.[10] Disability is defined under the Equality act of 2010 as having a physical or mental impairment that has substantial and long-term negative effect on the ability to do normal daily activities.[11] Despite the growing population of middle-aged and older adults and an increasing body of evidence suggesting sexual health is important to quality of life, there is limited evidence to support preferences and tailor services for specific groups within this population.[12,13]

This study will explore preferences for seeking sexual health services among middle-aged and older adults. This is part of a larger study to inform areas for improved sexual health


For peer review only - http://bmjopen.bmj.com/site/about/guidelines.xhtml

services for adults aged 45 and above, including disabled ones and from sexual minority groups living in the United Kingdom. We have chosen 45 years as our cut off age as menopause, erectile dysfunction and other sexual health issues become more common in that age group. Our study findings will explore issues related to service uptake and identify strategies to increase the uptake of sexual health services among adults aged 45 and above in the UK.

Discrete choice experiments (DCEs) are a quantitative approach to measure strength of preferences. It is an attribute-based measure of utility, based on the assumptions that healthcare interventions, services or policies, can be described by their attributes and that user preferences depend upon the levels of these attributes.[14] DCEs ask participants to make choices from hypothetical alternatives. They are increasingly used in health research to study patient and/or physician preferences.[15] For instance, DCEs have been used to determine preferences for HIV prevention and testing among a range of specific populations and settings to inform strategies for optimising health services.[16-21] DCEs have also been employed to study preferences for social care among older adults.[22] However, to date, no DCE studies have been conducted to determine preferences for sexual health services among older adults. Our study will use DCEs to explore preferences for accessing sexual health services and any heterogeneity that exists among middle-aged and older adults in the UK.

**Overall aim and objectives of this study**

The main aim of this study is to identify strategies to improve access of sexual health services among adults aged 45 and above including sexual minorities and disabled people, in the UK. Specific objectives include:

1) To identify barriers and facilitators for accessing sexual health services among adults aged 45 and above including sexual minorities and disabled people, in the UK using semi-structured interviews

2) To explore key preferences for accessing services for sexual health among middle-aged and older adults in the UK using a DCE survey

## Methods and Analysis

### Overview of approach and methods


For peer review only - http://bmjopen.bmj.com/site/about/guidelines.xhtml

The research will be conducted in two phases. First, we will organise semi-structured interviews with adults aged 45 or above to better understand their preferences and the importance of sexual health service and message sharing attributes in influencing their use of these services. Interview data will be analysed thematically, and emerging themes will inform which attributes and levels will be included in the choice sets for the DCEs.

**Patient and Public Involvement**

A key part of this study is community engagement as all studying findings will be dependent on response and input from the community members. With the aim of improving access to services, resulting strategies and solutions will be well received and more sustainable with sound community input. Consequently, community representatives have been recruited and engaged in the study design through local community-based organisations and reaching out through GP service. Community based organizations contacted where those specifically providing services to middle aged and older adults, disabled people and those from sexual minority groups. These will be continuously engaged in our survey distribution and participant recruitment, data interpretation, reporting, or dissemination plans of our research.

**Phase I- Semi-structured interviews**

DCEs are increasingly being recommended in health research to explore patient and public preferences.[22] This includes undertaking a conjoint analysis where related characteristics will be identified early on using literature reviews, group discussions or individual interviews.[23] First, we will conduct between 20 to 30 semi-structured individual interviews with adults aged 45 and above and stop when data saturation is reached. This formative qualitative research will identify factors that are considered in the decision-making processes among this population related to sexual health services (e.g., awareness, location, access process, quality of care or qualifications of healthcare providers, cost), identifying barriers and facilitators to service utilisation and sexual health message sharing based on older adults' perceptions of and/or experiences with sexual health services and interaction with sexual health messages. We will also field test the questionnaire using cognitive interviews prior to official start to help us understand how older individuals mentally process and respond to survey questions and DCE choice sets.

Participants will be purposively selected across different locations, age range, ethnic backgrounds, and disability status. Recruitment will be via community-based organisations.



For peer review only - http://bmjopen.bmj.com/site/about/guidelines.xhtml

We will create and disseminate a simple survey to collect data on profile including age, gender, ethnic background location in the UK, sexual orientation, relationship, and disability status. We will reach out to community-based organisations to disseminate this survey within their members and networks. From the responses received we will then contact participants purposively selected to ensure good spread and diversity in the sample recruited for interviews. Interviews will be conducted via telephone calls or teleconference using Zoom, Skype, or Microsoft Teams and facilitated by an experienced qualitative researcher. Teleconference calls may be either audio or video according to participant preferences. Interviews will be conducted using a topic guide (See Appendix I) and we will aim for approximately 30 minutes per interview session. Adjustments will be made as needed to promote the inclusion of disabled persons (e.g., use of sign language interpretation, simple language, inclusion of breaks). Each participant will receive an honorarium worth £30 in appreciation for their participation. A consent form will be provided to participants vie email, which will be signed and returned before the interview commences. Verbal consent will also be obtained right at the start of interview. Interviews will be audio-recorded following participant consent and transcribed verbatim prior to analysis.

**Qualitative data analysis**

We will undertake thematic analysis of the qualitative interview data using NVivo. A codebook will be developed both inductively and deductively to guide analysis. We will employ a framework approach and transcribe all interviews verbatim then commence coding of each transcript. Codes will be aggregated to build up the emerging themes. Outcomes of qualitative data analysis will be the indications and use of sexual health services, preferences and factors affecting their decision making to seek intimate and/or sexual relationships, barriers and facilitators to sexual health service utilisation. This qualitative data generated will be used to inform the DCE survey to be disseminated to participants in phase II. We will also disseminate the data as presentations in scientific conferences and publications in open access journals (See Appendix II for some preliminary results).

**Data Management**

All data (qualitative and quantitative) collected during the course of the research will be stored online and kept strictly confidential and will not be shared with anyone outside the research team. The identity of participants will be known by the researchers who will assign

For peer review only

participant numbers to anonymize responses. Responses will be de-identified prior to analysis, and results will be reported at an aggregate level, individual quotes when used will be followed by participant assigned numbers so that participants cannot be identified.

**Phase II- Discrete Choice Experiment survey**

Second, we will use the qualitative information from Phase I to inform what attributes (characteristics) and levels (choices) will be included in the unlabeled choice set, which will be administered in an online survey to a sample of older adults to identify their preferences for accessing sexual health services. We will design choice sets composed of hypothetical sexual health service delivery scenarios. Similarly, as stated for the interview, we will employ same strategy to recruit disabled people and people from sexual minority groups for the survey to allow sub-analyses of the results. We will aim to have disabled people and sexual minorities make up 10% each out of the total sample size.

**Developing the DCE design**

*Pictorial representation of attribute levels and DCE choice set size*

A ranking exercise will be organized with some of the interview participants to align and finalise the attributes and levels that will be included in the final choice sets (Appendix III). After attributes and levels have been finalised, we will generate visual graphics to represent each attribute level in the DCE survey (See Appendix IV for a sample). To ensure that the visual graphics effectively represent the intended meaning of each attribute level (i.e., construct validity), we will conduct qualitative think-aloud, pilot exercises with up to 10 individuals from the target population, including disabled people and those from sexual minority groups and participants will provide feedback about how they interpret and feel about the graphics.[24] This will be an iterative process with the draft pictorial concepts being tested until they are appropriately representing the intended concept. DCE surveys tend to present between 4 and 18 choice sets. During these interviews, we will also explore the optimal number of choice sets to present to each respondent to manage potential cognitive fatigue. Similar to the phase 1 interviews, participants will be purposively selected across different locations, age range, ethnic backgrounds and disability status. Again, reasonable accommodation will be made to promote inclusion of disabled persons, although people with severe cognitive/intellectual impairment will not be included in this step. Recruitment will be



For peer review only - http://bmjopen.bmj.com/site/about/guidelines.xhtml

through collaborations with community-based organisations with which our research team already have existing research collaborations with.

*Experimental design*

After visual graphics of the attribute levels have been finalised, we will use the software NGENE to develop the experimental design matrix for the DCE pilot.[25] For the pilot test, no assumptions about the parameter estimates will be made. We will then specify the appropriate number of attributes, levels, alternatives, blocks, and choice sets, and NGENE will iteratively generate design matrices of increasing statistical efficiency. We will use the design with the lowest D-error generated by NGENE. An example of a choice set and DCE design is presented in Figure 1. Graphics will have alt-text enabled so that they can be accessed by people with visual impairments.

*Blocking and randomisation of choice set ordering*

Depending on the total number of choice sets in the DCE design, choice sets may be grouped into blocks. Respondents will then be randomly assigned to one of the blocks and asked to complete all choice sets in their block. We anticipate using between 4-10 blocks, with each block containing 4-10 choice sets. To guard against potential ordering effects, the order of the choice sets within each block will be randomised for each participant.

*Pilot testing*

We intend to use convenience sampling to recruit up to 30 participants for the pilot test. Participants in the pilot test will receive a £20 gift card for completing the survey. Conducting the pilot test will serve two purposes: (1) identify potential problems with the DCE and survey in general (e.g., cognitive fatigue or incoherent questions or lack of accessibility) and (2) generate priors (i.e., parameter estimates) that will be used to develop the design of the final full-scale DCE. We will analyse the pilot data with multinomial logit models (MNL), given the limited sample size. Priors from the pilot test will be input into NGENE to finalise and enhance the efficiency of the full-scale DCE design.

*Full-scale DCE survey*

Community-based organizations, local clinics, GP practices and older people care homes will be contacted to help distribute the online survey. Recruitment will be carried out purposively from urban and suburban areas, evenly spread across varying gender mix and



For peer review only - http://bmjopen.bmj.com/site/about/guidelines.xhtml

the middle age and older adults age groups. We will create formats for completing the DCE surveys both online and offline.

**Eligibility criteria:**

We will recruit persons who meet all of the following criteria:

1) Adult aged 45 years or older

2) Had the experience of accessing sexual reproductive health service', prior to this study

3) Resident in the UK for at least six months

4) Willing to consent to participation.

With consent, participants of the DCE will be entered into a draw where up to 20 participants will be randomly selected to receive a £100 gift card.

*Quality assessment*

It is possible that some **participants** known as "speeders" may try to complete the DCE survey as soon as possible without seriously considering the survey questions. "Speeders" will be identified as those who complete the survey at least 50% faster than the median time for survey completion among all valid survey respondents. We will then conduct a sensitivity analysis and determine whether or not the removal of identified speeders significantly alters the results.

We will also assess the possibility of order effect bias by introducing a dummy variable for the order of the choice set. We will test for left and right bias by including a constant for the left choice and examining the extent to which the left position of the alternatives in the choice sets influences the likelihood of being chosen. Comparable probabilities of the left and centre alternatives being selected would suggest that the absence of bias.

**Sample size calculation**

Given the absence of any DCE priors for sexual health preferences of older adults in the UK, we used the following parametric-based DCE sample size calculation formula

For peer review only

$$n \geq \frac{(1-p)}{rpa^2} \times \left[ (\Phi^{-1}\left(1-\frac{\alpha}{2}\right) \right]^2$$

**Equation 1: Parametric DCE sample size formula[26]**

Whereby $n$ is the minimum sample size needed to detect $p$, the assumed true population probability of selecting uptake of sexual health services over “opt-out”, and whereby $r$ is the number of choice sets that each individual completes, $a$ is the level of precision around the assumed population probability, $\Phi^{-1}$ is the inverse cumulative normal distribution function, and $\alpha$ is the significance level.[26] Conservatively assuming that the true population probability of sexual health service uptake is 0.5 and assuming that each participant will complete eight choice sets, we estimate that a sample of 191 participants will be needed to generate a point estimate ± 0.05 of the true population probabilities and with 95% confidence. However, to maintain power for two-group subgroup analyses, we will aim to recruit a total **240** participants including those from sexual minorities groups and disabled people.

**Statistical analysis**

We will use descriptive statistics to summarize the key sociodemographic characteristics of the study population. We will assess sexual health service preferences and preference heterogeneity using multinomial logit (MNL), latent class (LC) and mixed logit (MXL) models. While the MNL is the starting point for most discrete choice models, it suffers from some unrealistic assumptions, particularly around unobserved heterogeneity. The MXL model can accommodate unobserved heterogeneity. The MXL analysis will be conducted with1000 Halton draws and will assume normally distributed preference heterogeneity for each attribute level. We will assess for unobserved heterogeneity by examining the statistical significance of the standard deviations of the random parameters in the MXL model. We will calculate the relative importance and predicted probabilities of opt-out for select sexual health service scenarios. We will assess how sexual health service and message sharing preferences could vary among those willing and unwilling to use sexual health services by using interaction terms for sociodemographic characteristics.

We will perform latent class analyses to elucidate if there are unobserved groups of people with similar preferences for use of sexual health service.[27] First, we will conduct a latent class conditional logit model that includes individual characteristics and attribute level preferences.[27] Selection of individual characteristics will be determined by expert opinion and

For peer review only

findings from the formative qualitative interviews. Second, we will determine the optimal number of subgroups by comparing the BIC values of 2, 3, 4, 5, 6, and 7-class models and each models' interpretability and class size distribution.[27] Third, we will use descriptive statistics to characterise each class based on their members' preferences and characteristics. All analyses will use effects coding and be weighted to reflect the population of middle-aged and older adults in the UK.

*Attribute relative importance and probability of opt-out*

For each of the sex-class combinations, the relative importance of each attribute will be calculated by dividing the range of the parameter estimates of a given attribute by the summation of the ranges of all attributes.[28]

We will further illustrate potential preference heterogeneities by using equation 2 to calculate the predicted probabilities of selecting "opt-out" over select sexual health service scenarios for each sex-class combination. Specifically, the predicted probability of a participant choosing alternative *i* over alternative *j* can be estimated by dividing the exponentiated summation of coefficients for scenario *i* by the exponentiated summation of coefficients for scenario *j*.

$$\text{Pi} = \frac{e^{\beta x_i}}{\sum e^{\beta x_j}}$$

$$\text{Pi} = \frac{e^{\beta x_i}}{\sum e^{\beta x_j}}$$

**Equation 2: Predicted probability of selecting alternative *i* within a two-alternative choice set**

**Impact**

This study will improve our understanding of older people's preferences and factors important for sexual health services. Our data will be useful to develop sexual health services and programs tailored for older people. By engaging older adults, this study will raise awareness for the need to access sexual health services irrespective of age and also ultimately identify strategies co-created by the end-users to increase service uptake.

**Feasibility**

Our project is feasible as the team has expertise from multiple disciplines to examine sexual health among older adults with unique strengths in DCEs, disability studies, and sexual health research.



For peer review only - http://bmjopen.bmj.com/site/about/guidelines.xhtml

## Ethics and dissemination

Ethical approval was obtained from the Research and Ethics Committee at the London School of Hygiene and Tropical Medicine, United Kingdom (26134). Findings from this study will be disseminated widely to relevant stakeholders via scheduled meetings, webinars, presentations at conferences and in peer reviewed journal manuscripts.

## Authors' contributions

DW and JDT conceived the idea. DW oversees the study. EEK and SWP wrote the first draft of the protocol paper. EEK, EG and SB conducted the interviews, developed attributes and levels, and JK helped refine the DCE survey design. JJO, SWP, FTP and JDT provided additional advice on the discrete choice experiment design and will carry out the experiment design. TJ, CT, HK, TS and JDT provided expertise advice on improving inclusiveness and appropriateness of the protocol. All authors reviewed, provided constructive comments on improving the design and the manuscript. All authors have seen and approve the final version of this manuscript.

## Funding

The study is supported by Economic and Social Research Council, UK Research and Innovation (UKRI) [grant number: ES/T014547/1].

## Competing interests

None.

## References:

1. Ezhova I, Savidge L, Bonnett C, Cassidy J, Okwuokei A, Dickinson T. Barriers to older adults seeking sexual health advice and treatment: A scoping review. *International journal of nursing studies*. 2020;107:103566.
2. McGrath M, Lynch E. Occupational therapists' perspectives on addressing sexual concerns of older adults in the context of rehabilitation. *Disability and rehabilitation*. 2014;36(8):651-657.
3. UK N. Report looks at the Health of Baby Boomers. Accessed 17 October, 2022. www.nhs.uk/news/older-people/report-looks-at-the-health-of-the-baby-boomer-generation/

For peer review only

4. Aging BRIo. Sexually Transmitted Diseases in Older Adults. Accessed 17 October, 2022. : www.benrose.org/Resources/article-stds-older-adults.cfm
5. Brennan-Ing M, Seidel L, London AS, Cahill S, Karpiak SE. Service utilization among older adults with HIV: The joint association of sexual identity and gender. *Community-Based Research on LGBT Aging*. Routledge; 2017:166-196.
6. Tanton C, Geary RS, Clifton S, et al. Sexual health clinic attendance and non-attendance in Britain: findings from the third National Survey of Sexual Attitudes and Lifestyles (Natsal-3). *Sexually transmitted infections*. 2018;94(4):268-276.
7. Gott M, Hinchliff S, Galena E. General practitioner attitudes to discussing sexual health issues with older people. *Social science & medicine*. 2004;58(11):2093-2103.
8. Beaudreau SA, Van Moorleghem K, Dodd SM, Liou-Johnson V, Suresh M, Gould CE. Satisfaction with a vacuum constriction device for erectile dysfunction among middle-aged and older veterans. *Clinical Gerontologist*. 2021;44(3):307-315.
9. Organization WH. *Summary: World report on disability 2011*. 2011. 6099570705.
10. GOV.UK. Family Resources Survey: financial year 2020 to 2021. Accessed 14 June, 2022. https://www.gov.uk/government/statistics/family-resources-survey-financial-year-2020-to-2021/family-resources-survey-financial-year-2020-to-2021#disability-1
11. GOV.UK. Equality Act 2010. GOV.UK. Accessed 02 March 2022, 2022. https://www.gov.uk/definition-of-disability-under-equality-act-2010#:~:text=You're%20disabled%20under%20the,to%20do%20normal%20daily%20activities.
12. Lindau ST, Schumm LP, Laumann EO, Levinson W, O'Muircheartaigh CA, Waite LJ. A study of sexuality and health among older adults in the United States. *New England Journal of Medicine*. 2007;357(8):762-774.
13. Hinchliff S, Tetley J, Lee D, Nazroo J. Older adults' experiences of sexual difficulties: qualitative findings from the English Longitudinal Study on Ageing (ELSA). *The Journal of Sex Research*. 2018;55(2):152-163.
14. Ryan M, Gerard K. Using discrete choice experiments to value health care programmes: current practice and future research reflections. *Applied health economics and health policy*. 2003;2(1):55-64.
15. Quaife M, Terris-Prestholt F, Di Tanna GL, Vickerman P. How well do discrete choice experiments predict health choices? A systematic review and meta-analysis of external validity. *The European journal of health economics*. 2018;19(8):1053-1066.
16. Strauss M, George G, Lansdell E, et al. HIV testing preferences among long distance truck drivers in Kenya: a discrete choice experiment. *AIDS care*. 2018;30(1):72-80.
17. Ong JJ, Nwaozuru U, Obiezu-Umeh C, et al. Designing HIV Testing and Self-Testing Services for Young People in Nigeria: A Discrete Choice Experiment. *The Patient-Patient-Centered Outcomes Research*. 2021:1-12.
18. Schaffer EM, Gonzalez JM, Wheeler SB, Kwarisiima D, Chamie G, Thirumurthy H. Promoting HIV testing by men: a discrete choice experiment to elicit preferences and predict uptake of community-based testing in Uganda. *Applied health economics and health policy*. 2020:1-20.
19. Ong JJ, Nwaozuru U, Obiezu-Umeh C, et al. Designing HIV Testing and Self-Testing Services for Young People in Nigeria: A Discrete Choice Experiment. *The Patient-Patient-Centered Outcomes Research*. 2021;14(6):815-826.

For peer review only

20. Pan SW, Durvasula M, Ong JJ, et al. No place like home? Disentangling preferences for HIV testing locations and services among men who have sex with men in China. *AIDS and Behavior*. 2019;23(4):847-859.
21. Terris-Prestholt F, Mulatu A, Quaife M, et al. Using choice experiments to improve equity in access to socially marketed HIV prevention products. *Journal of choice modelling*. 2021;41:100319.
22. Ryan M, Netten A, Skåtun D, Smith P. Using discrete choice experiments to estimate a preference-based measure of outcome—an application to social care for older people. *Journal of health economics*. 2006;25(5):927-944.
23. Ryan M, Farrar S. Using conjoint analysis to elicit preferences for health care. *Bmj*. 2000;320(7248):1530-1533.
24. Aday LA, Cornelius LJ. *Designing and conducting health surveys: a comprehensive guide*. John Wiley & Sons; 2006.
25. Metrics C. NGENE 1.1. 2 User Manual and Reference Guide: the cutting edge in experimental design. *USA: Choice Metrics*. 2014;
26. de Bekker-Grob EW, Donkers B, Jonker MF, Stolk EA. Sample size requirements for discrete-choice experiments in healthcare: a practical guide. *The Patient-Patient-Centered Outcomes Research*. 2015;8(5):373-384.
27. Zhou M, Thayer WM, Bridges JF. Using latent class analysis to model preference heterogeneity in health: a systematic review. *Pharmacoeconomics*. 2018;36(2):175-187.
28. Hauber AB, González JM, Groothuis-Oudshoorn CG, et al. Statistical methods for the analysis of discrete choice experiments: a report of the ISPOR conjoint analysis good research practices task force. *Value in health*. 2016;19(4):300-315.

For peer review only

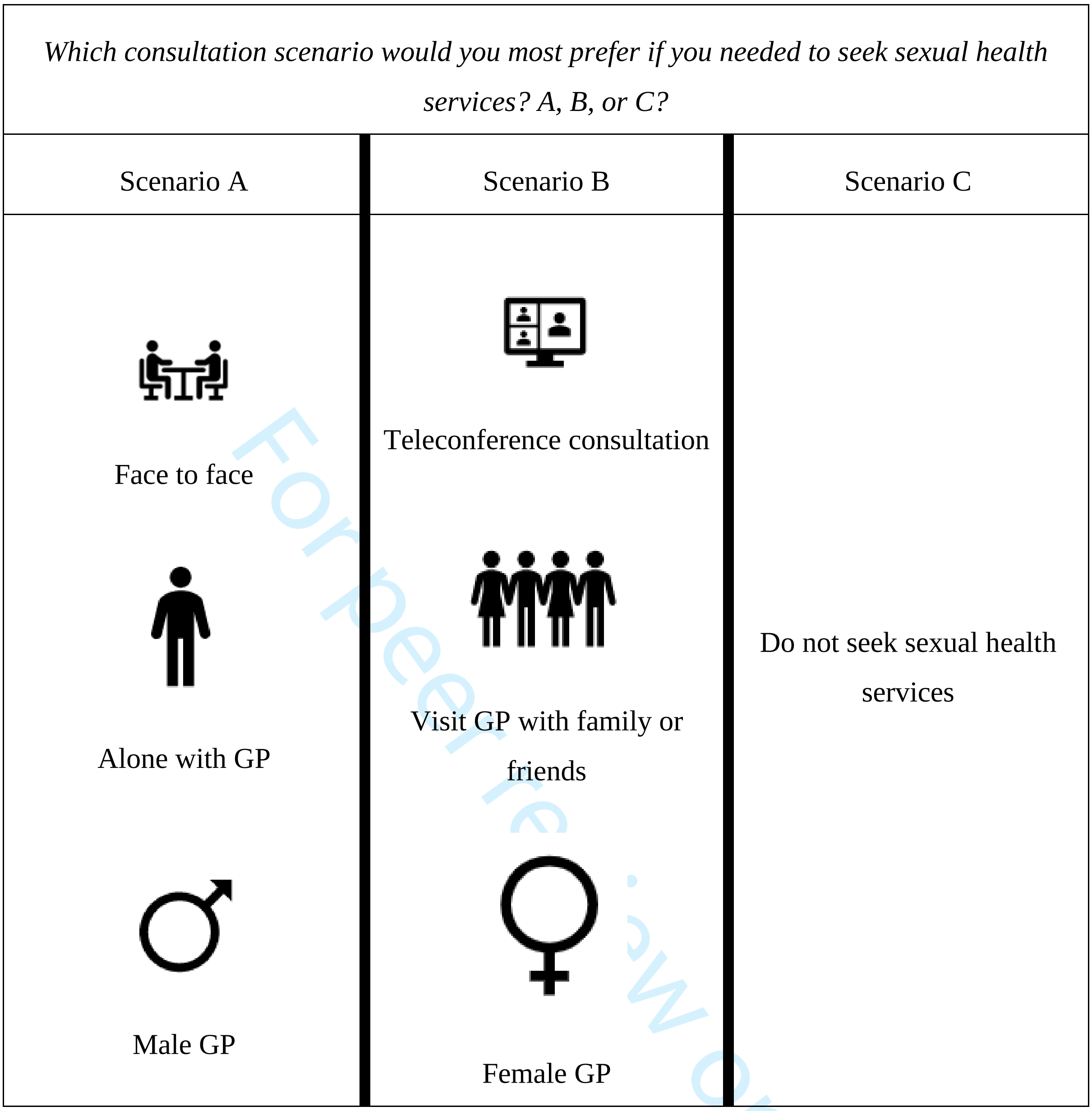


**Figure 1: Fictitious example of a choice set**

# Appendix I

## Sexual health in middle age and older adults in the UK
## Interview Topic Guide

Date of interview:

Respondent ID:

**Social Demographics**

Sex:

Age:

Ethnic group:

Local County:

Do you consider yourself to have a disability?

**Introduction (to be read by interviewer just before commencing the interview):**

Thank you for accepting to take part in this interview. I would just like to reiterate that everything you say in the interview is confidential. All data collected will be anonymized. The interview itself will be open ended and the questions themselves are usually broad. There aren't any right or wrong answers. I'm simply interested in your experience and your views about sexual health services. Sexual health is fundamental to the overall wellbeing of many individuals. Is there anything you'd like to ask me?

**Part I: Sexual health services**

1. Have you used sexual health services in the last (6-12months)?
2. If no, any reasons?
3. If yes, were you satisfied with the service received?
4. Overall, how would you rate sexual health services you received?
   <Very satisfied, Satisfied, Neutral, Dissatisfied, Very dissatisfied>
5. Do you feel sexual health services available to older adults are adequate?
6. Do you talk to your GP about sexual health concerns?
7. Does your GP bring up sexual health related discissions with you?
8. Do you feel comfortable posting or commenting on sexual health topics on social media?



For peer review only - http://bmjopen.bmj.com/site/about/guidelines.xhtml

**Part II: Decision making process**

1. What factors impact on your decision to seek sexual health services?
2. What would you access sexual health services for? General information and advice? Sexual function? STI testing?
3. How would you prefer to access sexual health services? Face to face or via telephone consultation? Or just online information?
4. For sexual health issues would you prefer to see a doctor that is:
    a. Same sex as you?
    b. Same ethnic background as you? Or speaks same language as you?
    c. A junior doctor or Very senior/ advanced doctor
    d. Have a friend, relation, partner go with you?
5. Where would you rather go for sexual health services? GP, Hospital, Pharmacy, Online, Older people groups/organisations medical outreach?

**Part III: Barriers and facilitators**

1. What factors prevent you from accessing sexual health services?
2. How would you prefer to access sexual health services?
3. What factors would make sexual health services easy and comfortable for you?
4. Would you be willing to participate in an open call and share messages about sexual health? With whom might you consider or not consider sharing the messages with? If so, what possible means of sharing would you prefer?

For peer review only

**Appendix II: Preliminary qualitative results**

So far, we have recruited and conducted 9 participants for the qualitative interviews as part of the first phase for the DCE project. Below are some preliminary results.

***Socio-demographic characteristics of participants***

There was a fair balance in gender with 4 males and 5 female respondents. Respondents were aged between 45 and 73 years with an average age of 58 years. All respondents were heterosexuals with more than half in a married /civil relationship (n=5). Few others were living with partner (n=2) and either single (n=1) or divorced (n=1). Respondents were either white-British (n=4) or black/African/British/Caribbean (n=5). Only one participant reported living with a disability and two-thirds of respondents confirmed using sexual health services within the last one year. See Table 1 below:

**Table 1: Socio-demographics of participants**

| **Characteristics** (n = 9) | **n (%)** |
|---|---|
| **Gender** | |
| Male | 4 (44) |
| Female | 5 (56) |
| **Age** (years) | |
| 45-50 | 3 (66) |
| 51-60 | 2 (22) |
| 61-70 | 3(33) |
| > 70 | 1 (11) |
| **Relationship status** | |
| Married/civil relationship | 5 (56) |
| Single | 1(11) |
| Living with partner | 2 (22) |
| Divorced | 1 (11) |
| **Sexual orientation** | |
| Straight heterosexual | 9 (100) |
| Homosexual gay/lesbian | 0 (0) |
| Bi-sexual | 0 (0) |
| **Ethnicity** | |
| White-British | 4 (44) |
| Black/British/Afro/Caribbean | 5 (56) |
| **Disability** | |
| Yes | 8 (89) |
| No | 1 (11) |
| **Used SH Service in last year?** | |
| Yes | 6 (67) |
| No | 3 (33) |



For peer review only - http://bmjopen.bmj.com/site/about/guidelines.xhtml

*Preferences for accessing sexual health services and sharing messages*

Participants that had used sexual health services were generally satisfied with services received. Most persons would rather not speak to their GP about their sexual health concerns unless it was absolutely necessary. Community sexual health clinics are preferred over GP's. Many older adults have sexual health needs related to decline sexual function and will not share sexual health related messages openly but are willing to send individual messages privately. Clear information about availability of sexual health services is needed.

**Table 2: Preferences for accessing sexual health services and sharing messages**

| **Questions** | **Responses** | **Themes** |
|---|---|---|
| Do you talk to your GP about sexual health concerns | No/ if there is a need to | Most persons do not feel comfortable talking to their regular GP about their sexual health needs |
| Would you feel comfortable sharing messages relating to sexual health topics on social media? | Majority – No<br>Can send message privately to family friend if needed | Respondents will not share sexual health related messages openly but are willing to send individual messages privately if there is a need |
| How would you prefer to access sexual health services? | Mixed responses- Face to face or online | Depending on the severity of the sexual health problem either face to face or an initial telephone/online consultation is preferred |
| What would you access sexual health services for? | Sexual function<br>STI testing<br>Contraception<br>General info/advice<br>Pre-menopausal concerns<br>Assisted reproduction | Many older adults have sexual health needs related to decline sexual function<br><br>There is need for clearer information on what services are available and how to access them |
| Where would you rather go for sexual health services? | Community NHS clinics, Not GP<br>Anonymous NHS staff<br>Online? | Community sexual health clinics are preferred over GP's. |
| What factors would you consider when accessing sexual health services? | Convenience, quality of service (professional), anonymity, discretion, | With sexual health quality and professional services is always key. A platform that is easy and convenient is also important |



For peer review only - http://bmjopen.bmj.com/site/about/guidelines.xhtml

## Appendix III: Draft Attributes and levels for ranking

| Attributes | Levels |
|---|---|
| **Service Type** | |
| *Service provider for consultation* | • GP clinic<br>• Sexual health clinic<br>• Pharmacy<br>• Online Platform |
| *Mode of delivery* | • Face to face<br>• Telephone call<br>• Video conference<br>• Online chat function |
| *Provider initiates discussion of sexual health* | • Yes<br>• No |
| *Anonymity* | • Name-based<br>• Partially anonymous<br>• Totally anonymous |
| **Provider Characteristics** | |
| *Gender ethnicity and age of health personnel* | • Same gender, age range and ethnicity<br>• Different gender age range and ethnicity |
| *Seniority of health personnel* | • Fairly recently qualified provider<br>• Older, more established provider |
| *Relationship with health personnel* | • Familiar provider/ existing relationship<br>• New provider |
| *Personnel with intersectional training (incl language on gender and sexual minorities, disabilities etc)* | • With intersectional training<br>• Without intersectional training |
| **Service Experience** | |
| *Who attends consultation with you* | • Family, friend, or personal care aide<br>• Medical assistant<br>• No one |
| *Provider privacy policy* | • Reassures patient of privacy<br>• No specific mention of privacy |
| *Accessibility* | • Accessible buildings and disability-friendly equipment<br>• Older, not updated accessibility accommodations |

For peer review only

## Appendix IV: Sample DCE survey

**Participant ID:**

Please do not leave any blank questions. There are no wrong answers.

**ELIGIBILITY**

01. Are you currently 45 years or older?

1) ☐ Yes、2) ☐ No

02. Have you had sexual health problems at all in the past five years?

1) ☐ Yes、2) ☐ No

03. How would you rate your sexual satisfaction in the past five years?

1) ☐ Very good、2) ☐ Good、3) ☐ Fair、4) Poor

04. How long have you been living in the UK?

1) ☐ Less than 6 months、2) ☐ Greater than 6 months、3) ☐ Not currently living in the UK

**A. DEMOGRAPHICS**

**A1.** What is your current age：______years old?

**A2.** What is your current sex?

1) Male

2) Female

**A1.** What city are you currently living in?

**A2.** What is your highest level of education?

1) ☐ Elementary
2) ☐ Middle school
3) ☐ High school
4) ☐ vocational college
5) ☐ 4-year college
6) ☐ graduate school

**A3.** What is your current marital/relationship status (past 6 months)?



For peer review only - http://bmjopen.bmj.com/site/about/guidelines.xhtml

1) ☐ Single, no significant partner
2) ☐ Currently living with significant partner
3) ☐ Married
4) ☐ Divorced
5) ☐ Widowed
7）☐ Other：________

**A4.** Would you be willing to use sexual health services?
1) Yes
2) No

**A5.** How would you rate your health in general (past 6 months)
1) ☐ excellent、2) ☐ very good、3) ☐ good、4) ☐ fair、5) ☐ poor

## B. Medical history & healthcare utilisation

B1. Have you ever been told by a healthcare professional that you have heart disease, cancer, diabetes, ADHD, a learning disability, asthma, chronic pain, or other chronic disease?
1) ☐ Yes、2) ☐ No [skip to D4]

B2. **If yes**, which one(s)? Check all that apply.
1) ☐ Heart disease、2) ☐ Cancer、3) ☐ Diabetes、4) ☐ ADHD/learning disability、
5) ☐ Asthma、6) ☐ chronic pain、7) ☐ Other [go to D3, otherwise skip to D4]

B3. **If other,** what was it? ______________

B4. In the past 2 years, how often have you visited a doctor?

1) ☐ I have not visited a doctor in the past 2 years、2) ☐ 1-2 times per year、3) ☐ 1-2 times per half-year、4) ☐ 1-2 times per month、5) ☐ 1-2 times per week

B5. In the past 2 years, have you ever avoided going to the doctor even though you were ill or injured?

1) ☐ Yes、2) ☐ No

B6. What type of health insurance do you have?

1) ☐ None、2) ☐ Urban employee、3) ☐ Urban resident、4) ☐ New rural cooperative、5) ☐ Private

B7. Would you be interested in discussing spiritual issues with your healthcare provider?


For peer review only - http://bmjopen.bmj.com/site/about/guidelines.xhtml

1) ☐ Yes、2) ☐ No、3) ☐ Unsure

B8. Do you have a (physical?) disability?

B9. If so, why type of (physical) disability?

B10. Have you ever consulted a healthcare provider for sexual health services (e.g., impotence, STIs, genital pain)?

## C: Sexual behaviours

C1. What is your sexual orientation?

1) ☐ Straight、2) ☐ Gay/homosexual、3) ☐ Bisexual、4) ☐ Other/uncertain

C2. Are you currently in a romantic sexual relationship?

1) ☐ Yes、2) ☐ No

C3. Have you ever had anal/oral/vaginal sex with another person?

1) ☐ Yes、2) ☐ No

C4. How old were you when you first had anal, oral, or vaginal sex? ___________ years old

C5. How many individuals have you ever had anal, oral, or vaginal sex with?

1) ☐ 1、2) ☐ 2-3、3) ☐ 4-5、4) ☐ 6-10、5) ☐ 11-20 、6) ☐ 21-50、7) ☐ >50

C6. Have you ever dated or had relations with anyone who has ever forced you to have sex when you did not want to？

1) ☐ Yes、2) ☐ No

C7. In the past 3 months, have you had sex? (check all that apply)

1) ☐ Yes, vaginal sex、2) ☐ Yes, anal sex、3) ☐ Yes, oral sex、4) ☐ No sex in past 3 months

C8. In the last 3 months, how often did you use a condom or other barrier method during sex?

1) ☐ Every time、2) ☐ Most of the time、3) ☐ Sometimes、4) ☐ Never

C9. Have any of these reasons kept you from getting tested for STI's (sexually transmitted infections)? If there's more than one reason, check all that apply

☐ You have never had sex
☐ It is too expensive
☐ You are afraid of what people might think
☐ You do not think you are at risk


For peer review only - http://bmjopen.bmj.com/site/about/guidelines.xhtml

- ☐ You do not want to know if you have something
- ☐ You do not know where to go to get tested
- ☐ You do not know what is involved in getting tested
- ☐ You do not know what it means to have a positive test
- ☐ Other (Specify): ______________________________

## D: Sexual health services Discrete choice Experiment

***The following questions will present three sexual health service scenarios (Choice 'A' and 'B'), and a "Do not seek sexual health services" scenario (Choice 'C').*** *Assuming that you have a need for sexual health services [examples or definitions inserted here] , which sexual health doctor consultation scenario would you most prefer? A,B, or C?* ***. There are no incorrect answers."***

D1. Please choose the scenario that you prefer the most

| Scenario A | Scenario B | Scenario C |
|---|---|---|
| 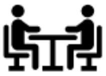<br>Face to face<br>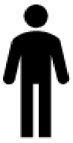<br>Alone with GP<br>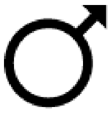<br>Male GP | 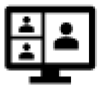<br>Teleconference consultation<br>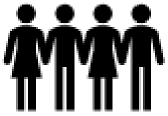<br>Visit GP with family or friends<br>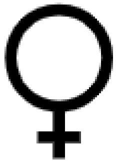<br>Female GP | Do not seek sexual health services |

D2. Please choose the scenario that you prefer the most

…

D3. Please choose the scenario that you prefer the most

…

D4. Please choose the scenario that you prefer the most

…

D5. Please choose the scenario that you prefer the most

…

D6. Please choose the scenario that you prefer the most



For peer review only - http://bmjopen.bmj.com/site/about/guidelines.xhtml

…

D7. Please choose the scenario that you prefer the most

…

D8. Please choose the scenario that you prefer the most

…

For peer review only

# Preferences for accessing sexual health services among middle-aged and older adults in the United Kingdom: a study protocol for a discrete choice experiment using mixed methods

| | |
|---|---|
| Journal: | *BMJ Open* |
| Manuscript ID | bmjopen-2022-066783.R3 |
| Article Type: | Protocol |
| Date Submitted by the Author: | 06-Apr-2023 |
| Complete List of Authors: | Kpokiri, Eneyi; London School of Hygiene and Tropical Medicine Faculty of Infectious and Tropical Diseases, Department of Clinical Research<br>Pan, Stephen W.; Xi'an Jiaotong-Liverpool University, Department of Health and Environmental Sciences<br>Ong, Jason; Monash University; London School of Hygiene & Tropical Medicine<br>Greaves, Emily; London School of Hygiene & Tropical Medicine<br>Khan, Junead; London School of Hygiene & Tropical Medicine<br>Bowen, Sophie; London School of Hygiene and Tropical Medicine Faculty of Infectious and Tropical Diseases, Department of Clinical Research<br>Jannaway, Tracey; Independent Living Alternatives<br>Terris-Prestholt, Fern; London School of Hygiene and Tropical Medicine<br>Tanton, Clare; London School of Hygiene and Tropical Medicine, Global Health & Development<br>Kuper, Hannah; The London School of Hygiene & Tropical Medicine, Clinical Research<br>Shakespeare, Tom; London School of Hygiene and Tropical Medicine Faculty of Infectious and Tropical Diseases<br>Tucker, Joseph; University of North Carolina Project China; London School of Hygiene and Tropical Medicine Faculty of Infectious and Tropical Diseases, Department of Clinical Research<br>Wu, Dan; London School of Hygiene and Tropical Medicine Faculty of Infectious and Tropical Diseases, Department of Clinical Research |
| <b>Primary Subject Heading</b>: | Sexual health |
| Secondary Subject Heading: | Geriatric medicine, Health services research |
| Keywords: | Sexual dysfunction < UROLOGY, SEXUAL MEDICINE, GERIATRIC MEDICINE |
| | |

For peer review only - http://bmjopen.bmj.com/site/about/guidelines.xhtml

# Preferences for accessing sexual health services among middle-aged and older adults in the United Kingdom: a study protocol for a discrete choice experiment using mixed methods

*Eneyi E. Kpokiri[1], *Stephen W. Pan[2], Jason J. Ong[1,3], Emily Greaves[1], Junead Khan[1], Sophie Bowen[1], Tracey Jannaway[4], Fern Terris-Prestholt[5], Clare Tanton[5], Hannah Kuper[1], Tom Shakespeare[1], Joseph D. Tucker[1,6], Dan Wu[1,7]

[1] Department of Clinical Research, London School of Hygiene and Tropical Medicine, London, United Kingdom

[2] Department of Health and Environmental Sciences, Xi'an Jiaotong-Liverpool University, Suzhou, China.

[3] Central Clinical School, Monash University, Melbourne, Australia

[4] Independent Living Alternatives, London, United Kingdom

[5] Faculty of Public Health & Policy, London School of Hygiene & Tropical Medicine, London, United Kingdom

[6] Division of Infectious Diseases, Department of Medicine, University of North Carolina at Chapel Hill, Chapel Hill, NC, United States

[7] Department of Epidemiology, West China School of Public Health, Sichuan University, Chengdu, China

*These authors contributed equally

Corresponding author:

Dan Wu, Department of Clinical Research, London School of Hygiene and Tropical Medicine, London, United Kingdom

Email: dan.wu@lshtm.ac.uk

## ABSTRACT

For peer review only

**Introduction:** Sexual health is essential for general health and wellbeing. Sexual health services for middle-aged and older adults are not prioritized and optimizing available services for this population is often overlooked. Not much is known about preferences for accessing sexual health services among middle-aged and older people or level of satisfaction with current services. The aim of this study is to explore preferences for seeking sexual health services among middle-aged and older adults in the United Kingdom (UK). The study will utilise Discrete Choice Experiments (DCEs) including initial qualitative interviews followed by the survey, which have been used as a tool to explore preferences in various health service delivery.

**Methods and Analysis:** The project will be carried out in two phases. First, we will conduct in-depth semi structured interviews with 20-30 adults (aged 45+), including disabled people, and those from sexual minority groups resident in the UK. Interviews will explore indications, preferences and factors related to accessing sexual health services. Themes and sub-themes emerging from the analysis of the interviews will then be used to design the choice sets and attribute level for the DCEs. For the second phase, for the DCEs, we will design choice sets composed of sexual health service delivery scenarios. The software NGENE will be used to develop the experimental design matrix for the DCE. We will use descriptive statistics to summarize the key sociodemographic characteristics of the study population. Multinomial logit, latent class and mixed logit models will be explored to assess sexual health service preferences and preference heterogeneity.

**Ethics and Dissemination:** Ethical approval for both parts of this study was granted by the Research and Ethics Committee at the London School of Hygiene and Tropical Medicine. Findings from this study will be disseminated widely to relevant stakeholders via scheduled meetings, webinars, presentations and journal publications.

For peer review only

**Strengths and Limitations of this study**

- This is the first DCE exploring preferences for sexual health services among middle-aged and older adults including disabled people.
- Our DCE is designed based on detailed formative research whereby choice sets are developed from emerging themes from 21 semi-structured interviews
- While the survey targets mature adults (age 45 and older), we sought to ensure inclusion of those from sexual minority groups and disabled people by recruiting from


For peer review only - http://bmjopen.bmj.com/site/about/guidelines.xhtml

local community-based organisations. This is to ensure diversity within this population to allow for sub-analysis

- Recruitment will be restricted to only adults aged 45 and older and living in the UK as a result, generalising the findings may be restricted to only the UK or other places with similar settings.

## Introduction

Sexual health is an integral part of health, wellbeing, and quality of life. However, many existing sexual health programmes, interventions and research are tailored for youth and younger adults.[1] The sexual health needs of middle-aged and older adults are often neglected, including by health professionals.[2] There are also a host of diverse challenges and barriers that interplay in how and why this age group access sexual health services. Older adults are increasingly experiencing higher rates of co-morbid illnesses further complicating their sexual health and well-being. Up to 9% increase in Sexually Transmitted Infections (STIs) among adults aged 50 and above have been reported. The rate of HIV infections is increasing faster among people aged 50 and above than in the under 40s, consequently, the number of people aged 50 and over living with HIV has now doubled in the last decade.[3,4] Older adults typically turn to family and friends to seek support for health needs and a study assessing sexual health clinic attendance in Britain found that non-attendance was higher among older adults. [5,6] Accordingly, there is suboptimal uptake of sexual health services among middle aged and older adults.[7,8] Within the broader group of "older people", certain groups may be particularly neglected, such as disabled older people, as they face additional barriers to accessing health services.[9] This is an important issue, as almost one in five people in the United Kingdom (UK) are aged 65+, and 42% of people in this age group report a disability.[10] Disability is defined under the Equality act of 2010 as having a physical or mental impairment that has substantial and long-term negative effect on the ability to do normal daily activities.[11] Despite the growing population of middle-aged and older adults and an increasing body of evidence suggesting sexual health is important to quality of life, there is limited evidence to support preferences and tailor services for specific groups within this population.[12,13]

This study will explore preferences for seeking sexual health services among middle-aged and older adults. This is part of a larger study to inform areas for improved sexual health services for adults aged 45 and above, including disabled ones and from sexual minority



For peer review only - http://bmjopen.bmj.com/site/about/guidelines.xhtml

groups living in the United Kingdom. We have chosen 45 years as our cut off age as menopause, erectile dysfunction and other sexual health issues become more common in that age group. Our study findings will explore issues related to service uptake and identify strategies to increase the uptake of sexual health services among adults aged 45 and above in the UK.

Discrete choice experiments (DCEs) are a quantitative approach to measure strength of preferences. It is an attribute-based measure of utility, based on the assumptions that healthcare interventions, services or policies, can be described by their attributes and that user preferences depend upon the levels of these attributes.[14] DCEs ask participants to make choices from hypothetical alternatives. They are increasingly used in health research to study patient and/or physician preferences.[15] For instance, DCEs have been used to determine preferences for HIV prevention and testing among a range of specific populations and settings to inform strategies for optimising health services.[16-21] DCEs have also been employed to study preferences for social care among older adults.[22] However, to date, no DCE studies have been conducted to determine preferences for sexual health services among older adults. Our study will use DCEs to explore preferences for accessing sexual health services and any heterogeneity that exists among middle-aged and older adults in the UK.

**Overall aim and objectives of this study**

The main aim of this study is to identify strategies to improve access of sexual health services among adults aged 45 and above including sexual minorities and disabled people, in the UK. Specific objectives include:

1) To identify barriers and facilitators for accessing sexual health services among adults aged 45 and above including sexual minorities and disabled people, in the UK using semi-structured interviews

2) To explore key preferences for accessing services for sexual health among middle-aged and older adults in the UK using a DCE survey

**Methods and Analysis**

**Overview of approach and methods**



For peer review only - http://bmjopen.bmj.com/site/about/guidelines.xhtml

The research will be conducted in two phases. First, we will organise semi-structured interviews with adults aged 45 or above to better understand their preferences and the importance of sexual health service and message sharing attributes in influencing their use of these services. Interview data will be analysed thematically, and emerging themes will inform which attributes and levels will be included in the choice sets for the DCEs.

**Patient and Public Involvement**

A key part of this study is community engagement as all studying findings will be dependent on response and input from the community members. With the aim of improving access to services, resulting strategies and solutions will be well received and more sustainable with sound community input. Consequently, community representatives have been recruited and engaged in the study design through local community-based organisations and reaching out through GP service. Community based organizations contacted where those specifically providing services to middle aged and older adults, disabled people and those from sexual minority groups. These will be continuously engaged in our survey distribution and participant recruitment, data interpretation, reporting, or dissemination plans of our research.

**Phase I- Semi-structured interviews**

DCEs are increasingly being recommended in health research to explore patient and public preferences.[22] This includes undertaking a conjoint analysis where related characteristics will be identified early on using literature reviews, group discussions or individual interviews.[23] First, we will conduct between 20 to 30 semi-structured individual interviews with adults aged 45 and above and stop when data saturation is reached. This formative qualitative research will identify factors that are considered in the decision-making processes among this population related to sexual health services (e.g., awareness, location, access process, quality of care or qualifications of healthcare providers, cost), identifying barriers and facilitators to service utilisation and sexual health message sharing based on older adults' perceptions of and/or experiences with sexual health services and interaction with sexual health messages. We will also field test the questionnaire using cognitive interviews prior to official start to help us understand how older individuals mentally process and respond to survey questions and DCE choice sets.

Participants will be purposively selected across different locations, age range, ethnic backgrounds, and disability status. Recruitment will be via community-based organisations.



For peer review only - http://bmjopen.bmj.com/site/about/guidelines.xhtml

We will create and disseminate a simple survey to collect data on profile including age, gender, ethnic background location in the UK, sexual orientation, relationship, and disability status. We will reach out to community-based organisations to disseminate this survey within their members and networks. From the responses received we will then contact participants purposively selected to ensure good spread and diversity in the sample recruited for interviews. Interviews will be conducted via telephone calls or teleconference using Zoom, Skype, or Microsoft Teams and facilitated by an experienced qualitative researcher. Teleconference calls may be either audio or video according to participant preferences. Interviews will be conducted using a topic guide (See Appendix I) and we will aim for approximately 30 minutes per interview session. Adjustments will be made as needed to promote the inclusion of disabled persons (e.g., use of sign language interpretation, simple language, inclusion of breaks). Each participant will receive an honorarium worth £30 in appreciation for their participation. A consent form will be provided to participants vie email, which will be signed and returned before the interview commences. Verbal consent will also be obtained right at the start of interview. Interviews will be audio-recorded following participant consent and transcribed verbatim prior to analysis.

**Qualitative data analysis**

We will undertake thematic analysis of the qualitative interview data using NVivo. A codebook will be developed both inductively and deductively to guide analysis. We will employ a framework approach and transcribe all interviews verbatim then commence coding of each transcript. Codes will be aggregated to build up the emerging themes. Outcomes of qualitative data analysis will be the indications and use of sexual health services, preferences and factors affecting their decision making to seek intimate and/or sexual relationships, barriers and facilitators to sexual health service utilisation. This qualitative data generated will be used to inform the DCE survey to be disseminated to participants in phase II. We will also disseminate the data as presentations in scientific conferences and publications in open access journals (See Appendix II for some preliminary results).

**Data Management**

All data (qualitative and quantitative) collected during the course of the research will be stored and manged securely online using our institute's Open Data Kit (ODK) at the London School of Hygiene and Tropical Medicine(LSHTM). An ODK server hosted at LSHTM



For peer review only - http://bmjopen.bmj.com/site/about/guidelines.xhtml

allows researchers to submit de-identified data electronically from anywhere in the world to a safe and secure server environment that is hosted on our institutional IT infrastructure. The identity of participants will be known by the researchers who will assign participant numbers to anonymize responses. Responses will be de-identified prior to analysis, and results will be reported at an aggregate level, individual quotes when used will be followed by participant assigned numbers so that participants cannot be identified.

**Phase II- Discrete Choice Experiment survey**

Second, we will use the qualitative information from Phase I to inform what attributes (characteristics) and levels (choices) will be included in the unlabeled choice set, which will be administered in an online survey to a sample of older adults to identify their preferences for accessing sexual health services. We will design choice sets composed of hypothetical sexual health service delivery scenarios. Similarly, as stated for the interview, we will employ same strategy to recruit disabled people and people from sexual minority groups for the survey to allow sub-analyses of the results. We will aim to have disabled people and sexual minorities make up 10% each out of the total sample size.

**Developing the DCE design**

*Pictorial representation of attribute levels and DCE choice set size*

A ranking exercise will be organized with some of the interview participants to align and finalise the attributes and levels that will be included in the final choice sets (Appendix III). After attributes and levels have been finalised, we will generate visual graphics to represent each attribute level in the DCE survey (See Appendix IV for a sample). To ensure that the visual graphics effectively represent the intended meaning of each attribute level (i.e., construct validity), we will conduct qualitative think-aloud, pilot exercises with up to 10 individuals from the target population, including disabled people and those from sexual minority groups and participants will provide feedback about how they interpret and feel about the graphics.[24] This will be an iterative process with the draft pictorial concepts being tested until they are appropriately representing the intended concept. DCE surveys tend to present between 4 and 18 choice sets. During these interviews, we will also explore the optimal number of choice sets to present to each respondent to manage potential cognitive fatigue. Similar to the phase 1 interviews, participants will be purposively selected across


For peer review only - http://bmjopen.bmj.com/site/about/guidelines.xhtml

different locations, age range, ethnic backgrounds and disability status. Again, reasonable accommodation will be made to promote inclusion of disabled persons, although people with severe cognitive/intellectual impairment will not be included in this step. Recruitment will be through collaborations with community-based organisations with which our research team already have existing research collaborations with.

*Experimental design*

After visual graphics of the attribute levels have been finalised, we will use the software NGENE to develop the experimental design matrix for the DCE pilot.[25] For the pilot test, no assumptions about the parameter estimates will be made. We will then specify the appropriate number of attributes, levels, alternatives, blocks, and choice sets, and NGENE will iteratively generate design matrices of increasing statistical efficiency. We will use the design with the lowest D-error generated by NGENE. An example of a choice set and DCE design is presented in Figure 1. Graphics will have alt-text enabled so that they can be accessed by people with visual impairments.

*Blocking and randomisation of choice set ordering*

Depending on the total number of choice sets in the DCE design, choice sets may be grouped into blocks. Respondents will then be randomly assigned to one of the blocks and asked to complete all choice sets in their block. We anticipate using between 4-10 blocks, with each block containing 4-10 choice sets. To guard against potential ordering effects, the order of the choice sets within each block will be randomised for each participant.

*Pilot testing*

We intend to use convenience sampling to recruit up to 30 participants for the pilot test. Participants in the pilot test will receive a £20 gift card for completing the survey. Conducting the pilot test will serve two purposes: (1) identify potential problems with the DCE and survey in general (e.g., cognitive fatigue or incoherent questions or lack of accessibility) and (2) generate priors (i.e., parameter estimates) that will be used to develop the design of the final full-scale DCE. We will analyse the pilot data with multinomial logit models (MNL), given the limited sample size. Priors from the pilot test will be input into NGENE to finalise and enhance the efficiency of the full-scale DCE design.

*Full-scale DCE survey*



For peer review only - http://bmjopen.bmj.com/site/about/guidelines.xhtml

Community-based organizations, local clinics, GP practices and older people care homes will be contacted to help distribute the online survey. Recruitment will be carried out purposively from urban and suburban areas, evenly spread across varying gender mix and the middle age and older adults age groups. We will create formats for completing the DCE surveys both online and offline.

**Eligibility criteria:**

We will recruit persons who meet all of the following criteria:

1) Adult aged 45 years or older

2) Had the experience of accessing sexual reproductive health service', prior to this study

3) Resident in the UK for at least six months

4) Willing to consent to participation.

With consent, participants of the DCE will be entered into a draw where up to 20 participants will be randomly selected to receive a £100 gift card.

*Quality assessment*

It is possible that some **participants** known as "speeders" may try to complete the DCE survey as soon as possible without seriously considering the survey questions. "Speeders" will be identified as those who complete the survey at least 50% faster than the median time for survey completion among all valid survey respondents. We will then conduct a sensitivity analysis and determine whether or not the removal of identified speeders significantly alters the results.

We will also assess the possibility of order effect bias by introducing a dummy variable for the order of the choice set. We will test for left and right bias by including a constant for the left choice and examining the extent to which the left position of the alternatives in the choice sets influences the likelihood of being chosen. Comparable probabilities of the left and centre alternatives being selected would suggest that the absence of bias.

**Sample size calculation**


For peer review only - http://bmjopen.bmj.com/site/about/guidelines.xhtml

Given the absence of any DCE priors for sexual health preferences of older adults in the UK, we used the following parametric-based DCE sample size calculation formula

$$n \geq \frac{(1-p)}{rpa^2} \times \left[(\Phi^{-1}\left(1-\frac{\alpha}{2}\right)\right]^2$$

**Equation 1: Parametric DCE sample size formula[26]**

Whereby *n* is the minimum sample size needed to detect *p*, the assumed true population probability of selecting uptake of sexual health services over "opt-out", and whereby *r* is the number of choice sets that each individual completes, *a* is the level of precision around the assumed population probability, $\Phi^{-1}$ is the inverse cumulative normal distribution function, and α is the significance level.[26] Conservatively assuming that the true population probability of sexual health service uptake is 0.5 and assuming that each participant will complete eight choice sets, we estimate that a sample of 191 participants will be needed to generate a point estimate ± 0.05 of the true population probabilities and with 95% confidence. However, to maintain power for two-group subgroup analyses, we will aim to recruit a total **240** participants including those from sexual minorities groups and disabled people.

### Statistical analysis

We will use descriptive statistics to summarize the key sociodemographic characteristics of the study population. We will assess sexual health service preferences and preference heterogeneity using multinomial logit (MNL), latent class (LC) and mixed logit (MXL) models. While the MNL is the starting point for most discrete choice models, it suffers from some unrealistic assumptions, particularly around unobserved heterogeneity. The MXL model can accommodate unobserved heterogeneity. The MXL analysis will be conducted with1000 Halton draws and will assume normally distributed preference heterogeneity for each attribute level. We will assess for unobserved heterogeneity by examining the statistical significance of the standard deviations of the random parameters in the MXL model. We will calculate the relative importance and predicted probabilities of opt-out for select sexual health service scenarios. We will assess how sexual health service and message sharing preferences could vary among those willing and unwilling to use sexual health services by using interaction terms for sociodemographic characteristics.



For peer review only - http://bmjopen.bmj.com/site/about/guidelines.xhtml

We will perform latent class analyses to elucidate if there are unobserved groups of people with similar preferences for use of sexual health service.[27] First, we will conduct a latent class conditional logit model that includes individual characteristics and attribute level preferences.[27] Selection of individual characteristics will be determined by expert opinion and findings from the formative qualitative interviews. Second, we will determine the optimal number of subgroups by comparing the BIC values of 2, 3, 4, 5, 6, and 7-class models and each models' interpretability and class size distribution.[27] Third, we will use descriptive statistics to characterise each class based on their members' preferences and characteristics. All analyses will use effects coding and be weighted to reflect the population of middle-aged and older adults in the UK.

*Attribute relative importance and probability of opt-out*

For each of the sex-class combinations, the relative importance of each attribute will be calculated by dividing the range of the parameter estimates of a given attribute by the summation of the ranges of all attributes.[28]

We will further illustrate potential preference heterogeneities by using equation 2 to calculate the predicted probabilities of selecting "opt-out" over select sexual health service scenarios for each sex-class combination. Specifically, the predicted probability of a participant choosing alternative *i* over alternative *j* can be estimated by dividing the exponentiated summation of coefficients for scenario *i* by the exponentiated summation of coefficients for scenario *j*.

$$\mathrm{Pi} = \frac{e^{\beta x_i}}{\sum e^{\beta x_j}} \quad \mathrm{Pi} = \frac{e^{\beta x_i}}{\sum e^{\beta x_j}}$$

**Equation 2: Predicted probability of selecting alternative *i* within a two-alternative choice set**

**Impact**

This study will improve our understanding of older people's preferences and factors important for sexual health services. Our data will be useful to develop sexual health services and programs tailored for older people. By engaging older adults, this study will raise awareness for the need to access sexual health services irrespective of age and also ultimately identify strategies co-created by the end-users to increase service uptake.

For peer review only



For peer review only - http://bmjopen.bmj.com/site/about/guidelines.xhtml

**Feasibility**

Our project is feasible as the team has expertise from multiple disciplines to examine sexual health among older adults with unique strengths in DCEs, disability studies, and sexual health research.

**Ethics and dissemination**

Ethical approval was obtained from the Research and Ethics Committee at the London School of Hygiene and Tropical Medicine, United Kingdom (26134). Findings from this study will be disseminated widely to relevant stakeholders via scheduled meetings, webinars, presentations at conferences and in peer reviewed journal manuscripts.

**Authors' contributions**

DW and JDT conceived the idea. DW oversees the study. EEK and SWP wrote the first draft of the protocol paper. EEK, EG and SB conducted the interviews, developed attributes and levels, and JK helped refine the DCE survey design. JJO, SWP, FTP and JDT provided additional advice on the discrete choice experiment design and will carry out the experiment design. TJ, CT, HK, TS and JDT provided expertise advice on improving inclusiveness and appropriateness of the protocol. All authors reviewed, provided constructive comments on improving the design and the manuscript. All authors have seen and approve the final version of this manuscript.

**Funding**

The study is supported by Economic and Social Research Council, UK Research and Innovation (UKRI) [grant number: ES/T014547/1].

**Competing interests**

None.

**References:**

1. Ezhova I, Savidge L, Bonnett C, Cassidy J, Okwuokei A, Dickinson T. Barriers to older adults seeking sexual health advice and treatment: A scoping review. *International journal of nursing studies*. 2020;107:103566.

For peer review only

2. McGrath M, Lynch E. Occupational therapists' perspectives on addressing sexual concerns of older adults in the context of rehabilitation. *Disability and rehabilitation*. 2014;36(8):651-657.
3. UK N. Report looks at the Health of Baby Boomers. Accessed 17 October, 2022. www.nhs.uk/news/older-people/report-looks-at-the-health-of-the-baby-boomer-generation/
4. Aging BRIo. Sexually Transmitted Diseases in Older Adults. Accessed 17 October, 2022. : www.benrose.org/Resources/article-stds-older-adults.cfm
5. Brennan-Ing M, Seidel L, London AS, Cahill S, Karpiak SE. Service utilization among older adults with HIV: The joint association of sexual identity and gender. *Community-Based Research on LGBT Aging*. Routledge; 2017:166-196.
6. Tanton C, Geary RS, Clifton S, et al. Sexual health clinic attendance and non-attendance in Britain: findings from the third National Survey of Sexual Attitudes and Lifestyles (Natsal-3). *Sexually transmitted infections*. 2018;94(4):268-276.
7. Gott M, Hinchliff S, Galena E. General practitioner attitudes to discussing sexual health issues with older people. *Social science & medicine*. 2004;58(11):2093-2103.
8. Beaudreau SA, Van Moorleghem K, Dodd SM, Liou-Johnson V, Suresh M, Gould CE. Satisfaction with a vacuum constriction device for erectile dysfunction among middle-aged and older veterans. *Clinical Gerontologist*. 2021;44(3):307-315.
9. Organization WH. *Summary: World report on disability 2011*. 2011. 6099570705.
10. GOV.UK. Family Resources Survey: financial year 2020 to 2021. Accessed 14 June, 2022. https://www.gov.uk/government/statistics/family-resources-survey-financial-year-2020-to-2021/family-resources-survey-financial-year-2020-to-2021#disability-1
11. GOV.UK. Equality Act 2010. GOV.UK. Accessed 02 March 2022, 2022. https://www.gov.uk/definition-of-disability-under-equality-act-2010#:~:text=You're%20disabled%20under%20the,to%20do%20normal%20daily%20activities.
12. Lindau ST, Schumm LP, Laumann EO, Levinson W, O'Muircheartaigh CA, Waite LJ. A study of sexuality and health among older adults in the United States. *New England Journal of Medicine*. 2007;357(8):762-774.
13. Hinchliff S, Tetley J, Lee D, Nazroo J. Older adults' experiences of sexual difficulties: qualitative findings from the English Longitudinal Study on Ageing (ELSA). *The Journal of Sex Research*. 2018;55(2):152-163.
14. Ryan M, Gerard K. Using discrete choice experiments to value health care programmes: current practice and future research reflections. *Applied health economics and health policy*. 2003;2(1):55-64.
15. Quaife M, Terris-Prestholt F, Di Tanna GL, Vickerman P. How well do discrete choice experiments predict health choices? A systematic review and meta-analysis of external validity. *The European journal of health economics*. 2018;19(8):1053-1066.
16. Strauss M, George G, Lansdell E, et al. HIV testing preferences among long distance truck drivers in Kenya: a discrete choice experiment. *AIDS care*. 2018;30(1):72-80.
17. Ong JJ, Nwaozuru U, Obiezu-Umeh C, et al. Designing HIV Testing and Self-Testing Services for Young People in Nigeria: A Discrete Choice Experiment. *The Patient-Patient-Centered Outcomes Research*. 2021:1-12.
18. Schaffer EM, Gonzalez JM, Wheeler SB, Kwarisiima D, Chamie G, Thirumurthy H. Promoting HIV testing by men: a discrete choice experiment to elicit preferences and predict uptake of community-based testing in Uganda. *Applied health economics and health policy*. 2020:1-20.
19. Ong JJ, Nwaozuru U, Obiezu-Umeh C, et al. Designing HIV Testing and Self-Testing Services for Young People in Nigeria: A Discrete Choice Experiment. *The Patient-Patient-Centered Outcomes Research*. 2021;14(6):815-826.


For peer review only - http://bmjopen.bmj.com/site/about/guidelines.xhtml

20. Pan SW, Durvasula M, Ong JJ, et al. No place like home? Disentangling preferences for HIV testing locations and services among men who have sex with men in China. *AIDS and Behavior*. 2019;23(4):847-859.
21. Terris-Prestholt F, Mulatu A, Quaife M, et al. Using choice experiments to improve equity in access to socially marketed HIV prevention products. *Journal of choice modelling*. 2021;41:100319.
22. Ryan M, Netten A, Skåtun D, Smith P. Using discrete choice experiments to estimate a preference-based measure of outcome—an application to social care for older people. *Journal of health economics*. 2006;25(5):927-944.
23. Ryan M, Farrar S. Using conjoint analysis to elicit preferences for health care. *Bmj*. 2000;320(7248):1530-1533.
24. Aday LA, Cornelius LJ. *Designing and conducting health surveys: a comprehensive guide*. John Wiley & Sons; 2006.
25. Metrics C. NGENE 1.1. 2 User Manual and Reference Guide: the cutting edge in experimental design. *USA: Choice Metrics*. 2014;
26. de Bekker-Grob EW, Donkers B, Jonker MF, Stolk EA. Sample size requirements for discrete-choice experiments in healthcare: a practical guide. *The Patient-Patient-Centered Outcomes Research*. 2015;8(5):373-384.
27. Zhou M, Thayer WM, Bridges JF. Using latent class analysis to model preference heterogeneity in health: a systematic review. *Pharmacoeconomics*. 2018;36(2):175-187.
28. Hauber AB, González JM, Groothuis-Oudshoorn CG, et al. Statistical methods for the analysis of discrete choice experiments: a report of the ISPOR conjoint analysis good research practices task force. *Value in health*. 2016;19(4):300-315.

For peer review only

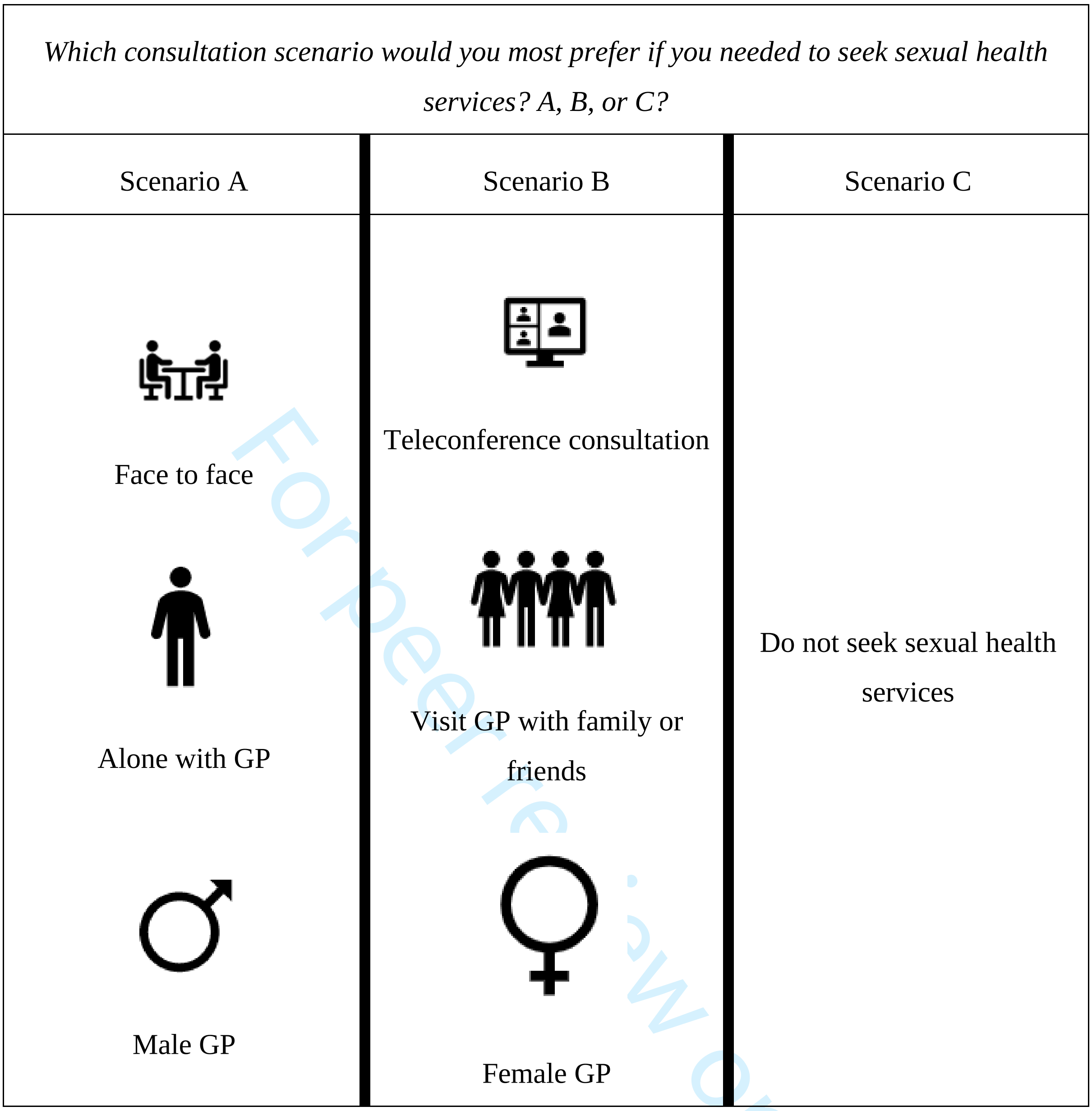


**Figure 1: Fictitious example of a choice set**

For peer review only

# Appendix I

## Sexual health in middle age and older adults in the UK
## Interview Topic Guide

Date of interview:

Respondent ID:

**Social Demographics**

Sex:

Age:

Ethnic group:

Local County:

Do you consider yourself to have a disability?

**Introduction (to be read by interviewer just before commencing the interview):**

Thank you for accepting to take part in this interview. I would just like to reiterate that everything you say in the interview is confidential. All data collected will be anonymized. The interview itself will be open ended and the questions themselves are usually broad. There aren't any right or wrong answers. I'm simply interested in your experience and your views about sexual health services. Sexual health is fundamental to the overall wellbeing of many individuals. Is there anything you'd like to ask me?

**Part I: Sexual health services**

1. Have you used sexual health services in the last (6-12months)?
2. If no, any reasons?
3. If yes, were you satisfied with the service received?
4. Overall, how would you rate sexual health services you received?
   <Very satisfied, Satisfied, Neutral, Dissatisfied, Very dissatisfied>
5. Do you feel sexual health services available to older adults are adequate?
6. Do you talk to your GP about sexual health concerns?
7. Does your GP bring up sexual health related discissions with you?
8. Do you feel comfortable posting or commenting on sexual health topics on social media?

For peer review only

**Part II: Decision making process**

1. What factors impact on your decision to seek sexual health services?
2. What would you access sexual health services for? General information and advice? Sexual function? STI testing?
3. How would you prefer to access sexual health services? Face to face or via telephone consultation? Or just online information?
4. For sexual health issues would you prefer to see a doctor that is:
    a. Same sex as you?
    b. Same ethnic background as you? Or speaks same language as you?
    c. A junior doctor or Very senior/ advanced doctor
    d. Have a friend, relation, partner go with you?
5. Where would you rather go for sexual health services? GP, Hospital, Pharmacy, Online, Older people groups/organisations medical outreach?

**Part III: Barriers and facilitators**

1. What factors prevent you from accessing sexual health services?
2. How would you prefer to access sexual health services?
3. What factors would make sexual health services easy and comfortable for you?
4. Would you be willing to participate in an open call and share messages about sexual health? With whom might you consider or not consider sharing the messages with? If so, what possible means of sharing would you prefer?

For peer review only

## Appendix II: Preliminary qualitative results

So far, we have recruited and conducted 9 participants for the qualitative interviews as part of the first phase for the DCE project. Below are some preliminary results.

***Socio-demographic characteristics of participants***

There was a fair balance in gender with 4 males and 5 female respondents. Respondents were aged between 45 and 73 years with an average age of 58 years. All respondents were heterosexuals with more than half in a married /civil relationship (n=5). Few others were living with partner (n=2) and either single (n=1) or divorced (n=1). Respondents were either white-British (n=4) or black/African/British/Caribbean (n=5). Only one participant reported living with a disability and two-thirds of respondents confirmed using sexual health services within the last one year. See Table 1 below:

**Table 1: Socio-demographics of participants**

| **Characteristics** (n = 9) | **n (%)** |
|---|---|
| **Gender** | |
| Male | 4 (44) |
| Female | 5 (56) |
| **Age** (years) | |
| 45-50 | 3 (66) |
| 51-60 | 2 (22) |
| 61-70 | 3(33) |
| > 70 | 1 (11) |
| **Relationship status** | |
| Married/civil relationship | 5 (56) |
| Single | 1(11) |
| Living with partner | 2 (22) |
| Divorced | 1 (11) |
| **Sexual orientation** | |
| Straight heterosexual | 9 (100) |
| Homosexual gay/lesbian | 0 (0) |
| Bi-sexual | 0 (0) |
| **Ethnicity** | |
| White-British | 4 (44) |
| Black/British/Afro/Caribbean | 5 (56) |
| **Disability** | |
| Yes | 8 (89) |
| No | 1 (11) |
| **Used SH Service in last year?** | |
| Yes | 6 (67) |
| No | 3 (33) |



For peer review only - http://bmjopen.bmj.com/site/about/guidelines.xhtml

*Preferences for accessing sexual health services and sharing messages*

Participants that had used sexual health services were generally satisfied with services received. Most persons would rather not speak to their GP about their sexual health concerns unless it was absolutely necessary. Community sexual health clinics are preferred over GP's. Many older adults have sexual health needs related to decline sexual function and will not share sexual health related messages openly but are willing to send individual messages privately. Clear information about availability of sexual health services is needed.

**Table 2: Preferences for accessing sexual health services and sharing messages**

| **Questions** | **Responses** | **Themes** |
|---|---|---|
| Do you talk to your GP about sexual health concerns | No/ if there is a need to | Most persons do not feel comfortable talking to their regular GP about their sexual health needs |
| Would you feel comfortable sharing messages relating to sexual health topics on social media? | Majority – No<br>Can send message privately to family friend if needed | Respondents will not share sexual health related messages openly but are willing to send individual messages privately if there is a need |
| How would you prefer to access sexual health services? | Mixed responses- Face to face or online | Depending on the severity of the sexual health problem either face to face or an initial telephone/online consultation is preferred |
| What would you access sexual health services for? | Sexual function<br>STI testing<br>Contraception<br>General info/advice<br>Pre-menopausal concerns<br>Assisted reproduction | Many older adults have sexual health needs related to decline sexual function<br><br>There is need for clearer information on what services are available and how to access them |
| Where would you rather go for sexual health services? | Community NHS clinics, Not GP<br>Anonymous NHS staff<br>Online? | Community sexual health clinics are preferred over GP's. |
| What factors would you consider when accessing sexual health services? | Convenience, quality of service (professional), anonymity, discretion, | With sexual health quality and professional services is always key. A platform that is easy and convenient is also important |



For peer review only - http://bmjopen.bmj.com/site/about/guidelines.xhtml

# Appendix III: Draft Attributes and levels for ranking

| Attributes | Levels |
|---|---|
| **Service Type** | |
| *Service provider for consultation* | • GP clinic<br>• Sexual health clinic<br>• Pharmacy<br>• Online Platform |
| *Mode of delivery* | • Face to face<br>• Telephone call<br>• Video conference<br>• Online chat function |
| *Provider initiates discussion of sexual health* | • Yes<br>• No |
| *Anonymity* | • Name-based<br>• Partially anonymous<br>• Totally anonymous |
| **Provider Characteristics** | |
| *Gender ethnicity and age of health personnel* | • Same gender, age range and ethnicity<br>• Different gender age range and ethnicity |
| *Seniority of health personnel* | • Fairly recently qualified provider<br>• Older, more established provider |
| *Relationship with health personnel* | • Familiar provider/ existing relationship<br>• New provider |
| *Personnel with intersectional training (incl language on gender and sexual minorities, disabilities etc)* | • With intersectional training<br>• Without intersectional training |
| **Service Experience** | |
| *Who attends consultation with you* | • Family, friend, or personal care aide<br>• Medical assistant<br>• No one |
| *Provider privacy policy* | • Reassures patient of privacy<br>• No specific mention of privacy |
| *Accessibility* | • Accessible buildings and disability-friendly equipment<br>• Older, not updated accessibility accommodations |

For peer review only

**Appendix IV: Sample DCE survey**

**Participant ID:**

Please do not leave any blank questions. There are no wrong answers.

**ELIGIBILITY**

01. Are you currently 45 years or older?

1) ☐ Yes、2) ☐ No

02. Have you had sexual health problems at all in the past five years?

1) ☐ Yes、2) ☐ No

03. How would you rate your sexual satisfaction in the past five years?

1) ☐ Very good、2) ☐ Good、3) ☐ Fair、4) Poor

04. How long have you been living in the UK?

1) ☐ Less than 6 months、2) ☐ Greater than 6 months、3) ☐ Not currently living in the UK

**A. DEMOGRAPHICS**

**A1.** What is your current age：______years old?

**A2.** What is your current sex?

1) Male

2) Female

**A1.** What city are you currently living in?

**A2.** What is your highest level of education?

1) ☐ Elementary
2) ☐ Middle school
3) ☐ High school
4) ☐ vocational college
5) ☐ 4-year college
6) ☐ graduate school

**A3.** What is your current marital/relationship status (past 6 months)?


For peer review only - http://bmjopen.bmj.com/site/about/guidelines.xhtml

1) ☐ Single, no significant partner
2) ☐ Currently living with significant partner
3) ☐ Married
4) ☐ Divorced
5) ☐ Widowed
7）☐ Other：________

**A4.** Would you be willing to use sexual health services?
1) Yes
2) No

**A5.** How would you rate your health in general (past 6 months)
1) ☐ excellent、2) ☐ very good、3) ☐ good、4) ☐ fair、5) ☐ poor

## B. Medical history & healthcare utilisation

B1. Have you ever been told by a healthcare professional that you have heart disease, cancer, diabetes, ADHD, a learning disability, asthma, chronic pain, or other chronic disease?
1) ☐ Yes、2) ☐ No [skip to D4]

B2. **If yes**, which one(s)? Check all that apply.
1) ☐ Heart disease、2) ☐ Cancer、3) ☐ Diabetes、4) ☐ ADHD/learning disability、
5) ☐ Asthma、6) ☐ chronic pain、7) ☐ Other [go to D3, otherwise skip to D4]

B3. **If other,** what was it? ______________

B4. In the past 2 years, how often have you visited a doctor?

1) ☐ I have not visited a doctor in the past 2 years、2) ☐ 1-2 times per year、3) ☐ 1-2 times per half-year、4) ☐ 1-2 times per month、5) ☐ 1-2 times per week

B5. In the past 2 years, have you ever avoided going to the doctor even though you were ill or injured?

1) ☐ Yes、2) ☐ No

B6. What type of health insurance do you have?

1) ☐ None、2) ☐ Urban employee、3) ☐ Urban resident、4) ☐ New rural cooperative、5) ☐ Private

B7. Would you be interested in discussing spiritual issues with your healthcare provider?


For peer review only - http://bmjopen.bmj.com/site/about/guidelines.xhtml

1) ☐ Yes、2) ☐ No、3) ☐ Unsure

B8. Do you have a (physical?) disability?

B9. If so, why type of (physical) disability?

B10. Have you ever consulted a healthcare provider for sexual health services (e.g., impotence, STIs, genital pain)?

## C: Sexual behaviours

C1. What is your sexual orientation?
1) ☐ Straight、2) ☐ Gay/homosexual、3) ☐ Bisexual、4) ☐ Other/uncertain

C2. Are you currently in a romantic sexual relationship?
1) ☐ Yes、2) ☐ No

C3. Have you ever had anal/oral/vaginal sex with another person?
1) ☐ Yes、2) ☐ No

C4. How old were you when you first had anal, oral, or vaginal sex? __________ years old

C5. How many individuals have you ever had anal, oral, or vaginal sex with?
1) ☐ 1、2) ☐ 2-3、3) ☐ 4-5、4) ☐ 6-10、5) ☐ 11-20 、6) ☐ 21-50、7) ☐ >50

C6. Have you ever dated or had relations with anyone who has ever forced you to have sex when you did not want to？
1) ☐ Yes、2) ☐ No

C7. In the past 3 months, have you had sex? (check all that apply)
1) ☐ Yes, vaginal sex、2) ☐ Yes, anal sex、3) ☐ Yes, oral sex、4) ☐ No sex in past 3 months

C8. In the last 3 months, how often did you use a condom or other barrier method during sex?
1) ☐ Every time、2) ☐ Most of the time、3) ☐ Sometimes、4) ☐ Never

C9. Have any of these reasons kept you from getting tested for STI's (sexually transmitted infections)? If there's more than one reason, check all that apply
☐ You have never had sex
☐ It is too expensive
☐ You are afraid of what people might think
☐ You do not think you are at risk

For peer review only

- ☐ You do not want to know if you have something
- ☐ You do not know where to go to get tested
- ☐ You do not know what is involved in getting tested
- ☐ You do not know what it means to have a positive test
- ☐ Other (Specify): ________________________________

## D: Sexual health services Discrete choice Experiment

***The following questions will present three sexual health service scenarios (Choice 'A' and 'B'), and a "Do not seek sexual health services" scenario (Choice 'C').*** *Assuming that you have a need for sexual health services [examples or definitions inserted here] , which sexual health doctor consultation scenario would you most prefer? A,B, or C?* **.** ***There are no incorrect answers."***

D1. Please choose the scenario that you prefer the most

| Scenario A | Scenario B | Scenario C |
|---|---|---|
| 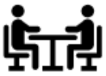 Face to face<br>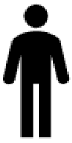 Alone with GP<br>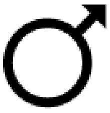 Male GP | 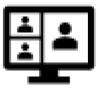 Teleconference consultation<br>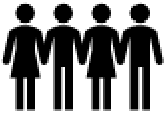 Visit GP with family or friends<br>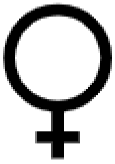 Female GP | Do not seek sexual health services |

D2. Please choose the scenario that you prefer the most

…

D3. Please choose the scenario that you prefer the most

…

D4. Please choose the scenario that you prefer the most

…

D5. Please choose the scenario that you prefer the most

…

D6. Please choose the scenario that you prefer the most

For peer review only - http://bmjopen.bmj.com/site/about/guidelines.xhtml

…
D7. Please choose the scenario that you prefer the most
…
D8. Please choose the scenario that you prefer the most
…

For peer review only
